# Computational Study on the Water Corrosion Process at Schreibersite (Fe_2_NiP) Surfaces: from Phosphide to Phosphates

**DOI:** 10.1021/acsearthspacechem.3c00167

**Published:** 2023-09-21

**Authors:** Stefano Pantaleone, Marta Corno, Albert Rimola, Nadia Balucani, Piero Ugliengo

**Affiliations:** † Dipartimento di Chimica and Nanostructured Interfaces and Surfaces (NIS) Centre, Università degli Studi di Torino, via P. Giuria 7,, I-10125 Torino, Italy; ‡ Dipartimento di Chimica, Biologia e Biotecnologie, Università degli Studi di Perugia, Via Elce di Sotto 8, I-06123 Perugia, Italy; § Departament de Química, Universitat Autònoma de Barcelona, 08193 Bellaterra, Catalonia, Spain; ∥ Osservatorio Astrofisico di Arcetri, Largo E. Fermi 5, I-50125 Firenze, Italy; ⊥ Université Grenoble Alpes, CNRS, Institut de Planétologie et d’Astrophysique de Grenoble (IPAG), F-38000 Grenoble, France

**Keywords:** meteorites, phosphorus problem, DFT, prebiotic chemistry, water corrosion

## Abstract

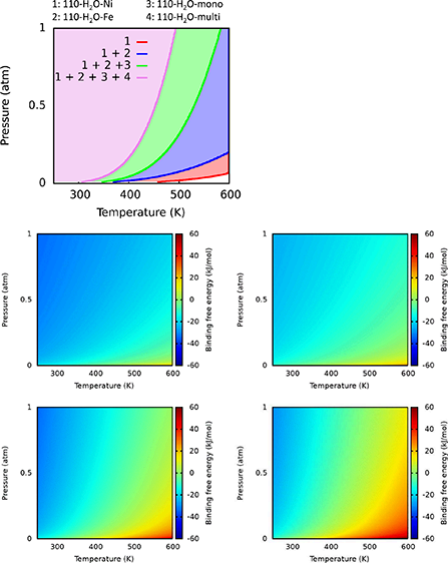

Phosphorus (P) is a fundamental element for whatever form of life, in the same way as the other biogenic macroelements (SONCH). The prebiotic origin of P is still a matter of debate, as the phosphates present on earth are trapped in almost insoluble solid matrixes (apatites) and, therefore, hardly available for inclusion in living systems in the prebiotic era. The most accepted theories regard a possible exogenous origin during the Archean Era, through the meteoritic bombardment, when tons of reactive P in the form of phosphide ((Fe,Ni)_3_P, schreibersite mineral) reached the primordial earth, reacting with water and providing oxygenated phosphorus compounds (including phosphates). In the last 20 years, laboratory experiments demonstrated that the corrosion process of schreibersite by water indeed leads to reactive phosphates that, in turn, react with other biological building blocks (nucleosides and simple sugars) to form more complex molecules (nucleotides and complex sugars). In the present paper, we study the water corrosion of different crystalline surfaces of schreibersite by means of periodic DFT (density functional theory) simulations. Our results show that water adsorbs molecularly on the most stable (110) surface but dissociates on the less stable (001) one, giving rise to further reactivity. Indeed, subsequent water adsorptions, up to the water monolayer coverage, show that, on the (001) surface, iron and nickel atoms are the first species undergoing the corrosion process and, in a second stage, the phosphorus atoms also get involved. When adsorbing up to three and four water molecules per unit cell, the most stable structures found are the phosphite and phosphate forms of phosphorus, respectively. Simulation of the vibrational spectra of the considered reaction products revealed that the experimental band at 2423 cm^–1^ attributed to the P–H stretching frequency is indeed predicted for a phosphite moiety attached to the schreibersite (001) surface upon chemisorption of up to three water molecules.

## Introduction

All living creatures, irrespective of being either the simplest monocellular forms of algae or the most complex mammals, share the same fundamental constituents and the same few molecular bricks that combine with each other to form an infinite variety of complex macromolecular systems: amino acids for proteins, lipids for the lipidic membranes of the cells, sugars, and nucleobases for DNA and RNA. These macromolecules are mostly formed by few macroelements (SONCH), to whom P deserves to be added because, although it represents only 1% w/w in terms of abundance with respect to the other macroelements, it is a key element in the form of phosphate (PO_4_^3–^) devoted to

- activating and deactivating the specific function of a protein,
- forming the cell membranes (phospholipids),
- connecting the nucleobases with each other, thus forming, together with ribose molecules, the outermost part of the double helix,^1^
- forming nucleotides essential for the bioenergetic of the cell.

The history of the simple molecular bricks—how they arrived on the primordial earth and their abundance nowadays—is still a matter of debate, but there are some points on which the scientific community generally agrees. In this respect, it is commonly accepted that meteorites have played the fundamental role of carriers of these crucial seeds of life, thanks to analysis performed on meteorites that fell on the earth^2−6^ or even *in loco* on orbiting comets.^7^

In the specific case of phosphorus, Gulick proposed that it came on earth mostly trapped in metal matrixes in the mineral form of schreibersite (Fe,Ni)_3_P,^8^ present in iron meteorites.^9−11^ After 50 years, this hypothesis became reality when, in 2005, Pasek et al. carried out the first corrosion experiment on synthetic schreibersite operated by water, in which different oxygenated phosphorus compounds were formed, without using catalysts or adopting high temperatures.^12^ In the following years, many other experiments were performed, where^13−19^ organic species necessary for life (nitrogenous bases and simple sugars) were added to the above-mentioned experiment.^13−19^ Results showed that not only is schreibersite capable of producing phosphates but also the reduced phosphorus contained within is reactive enough to activate phosphorylation reactions, producing nucleotides and complex sugars. The real problem, however, is not the lack of phosphorus on earth; indeed, it is very abundant in apatite minerals and already in the biological form of phosphate. However, apatites are neither reactive nor soluble, and the phosphate therein is not available for a phosphorylation reaction with specific prebiotic molecules.^20^

It is worth mentioning those studies dealing with the abundance of schreibersite on the early earth crust and, accordingly, the possible phosphate intake. Mathematical models show that a metallic based meteorite of about 60 tons produces ca. 1 ton of phosphorus (taking into account all the possible loose of material, such as during the contact with the terrestrial atmosphere) and that after the Late Heavy Bombardment in the Hadean era, 1–10% of the earth crust was composed by phosphide minerals.^21^ Another recent study showed a different route to obtain phosphorus oxygenated compounds that does not depend on the meteor bombardment flux or on the amount of phosphide contained in the meteors. Instead, it shows that schreibersite could be produced in situ by photoactivated reactions on clays.^22^ This would have ensured a continuous production of 10–1000 kg/year of phosphide and 100–10000 kg/year of phosphite and hypophosphite.^22^

Despite the many experimental studies carried out so far, a mechanistic analysis of the corrosion process of schreibersite is missing in the literature. Very few computational works are available on this kind of material; on Fe_3_P, i.e., the forerunner of bulk schreibersite, periodic quantum mechanical simulations were performed dedicated to predict its phase stability under the extreme conditions of temperature and pressure that can be found in meteorites and comets.^23−25^ In a recent work published by us, we studied and characterized bulk and surfaces of Fe_2_NiP, calibrating the methodology by comparing our simulation with experimental Raman spectra.^26^ In the last year, two papers studying single water adsorption and deprotonation on the most stable (110) surface of Fe_3_P and Fe_2_NiP were published,^27,28^ elucidating some features not clear from the experiments, i.e., on which sites water adsorbs (only on metals if it is in the molecular form), and the fact that water deprotonation is not thermodynamically favorable (at least on the most stable surface). A comparison with previous corrosion experiments^13,17^ followed by IR spectroscopy is also available showing a very good agreement between computed and experimental results.^27^

In the present paper, we focus our attention on the reactivity of water molecules on the top of both the (110) and the (001) surfaces of Fe_2_NiP (with more details on the less stable and more reactive one, i.e., the (001)) by means of periodic DFT simulations through the use of the plane-waves based code VASP. The difference in terms of stability of these two surfaces as well as the effect they have on the formation reaction of phosphonic (H_3_PO_3_) and phosphoric (H_3_PO_4_) acids is discussed.

## Computational Details

The adsorption of water on the (001) (composed by 24 formula units) and (110) (composed by 12 formula units) crystal faces of bulk schreibersite (composed by 8 formula units) was studied by means of periodic DFT calculations carried out with the Vienna Ab initio Simulation Package (VASP) code,^29−32^ which uses projector-augmented wave (PAW) pseudopotentials^33^ to describe the ionic cores and a plane wave basis set for the valence electrons. The same approach was used in our previous work^26^ to characterize the pristine schreibersite (both the bulk and the (110) and (001) surface structures).

Geometry optimizations and frequency calculations were performed with the gradient corrected PBE functional,^34^ with a posteriori Grimme D2 correction,^35^ modified for solids (D*).^36^ Moreover, C_6_ atomic coefficients related to polarizabilities on Fe and Ni metal atoms were set to 0 (i.e., no dispersion interaction contribution from metal atoms). This setup was chosen according to the best results obtained in our previous work on the bulk and bare surfaces of schreibersite.^26^ On O and H atoms, the original D* parameters were used. This method of choice is termed PBE-D*0 in this the work. The cutoff energy of plane waves (which controls the accuracy of the calculations) was set to 500 eV. The self-consistent field (SCF) iterative procedure was converged to a tolerance in total energy of Δ*E* = 10^–5^ eV for geometry optimizations, whereas for frequency calculations the tolerance was decreased to Δ*E* = 10^–6^ eV. The tolerance on gradients during the optimization procedure was set to 0.01 eV/Å for each atom in each direction. On specific interesting structures of relevance from the point of view of the reactivity, geometry optimizations were also carried out in water polarizable continuous solvent (PCM). The (110) unit cell was enlarged to a 2 × 2 supercell (*a* = 8.749 Å, *b* = 13.437 Å, γ = 108.998°) to ensure enough space for the reactivity of water, whereas the (001) was used as it is (*a* = 8.984 Å, *b* = 8.984 Å, γ = 90.000°); here we remind that the (001) unit cell contains twice the number of atoms of the (110). The Monkhorst–Pack sampling of the Brillouin zone was used for the *k*-point mesh. Shrinking factors for both (110) and (001) surfaces have been set to (4 4 1), the last number being related to the nonperiodic direction (i.e., no sampling of the reciprocal space). As VASP relies on plane waves basis set and, accordingly, surfaces are replicated also along the nonperiodic direction, the vacuum space among fictitious replicas was set to at least 20 Å to minimize the interactions among replica images. Therefore, the final *c* cell axis was set to 40 and 35 Å for the (110) and (001) surfaces, respectively, as the slab thickness is 3–4 Å larger for (110). Geometry optimizations were carried out by moving all atoms in the unit cell while keeping the cell parameters fixed at the optimized geometry of the bulk to enforce the rigidity experienced by a macroscopic surface (instead of a thin film model). The only simulations in which also the cell parameters were set free to relax are those related to the water multilayer to allow the system to reach the correct liquid water density.

Adsorption energies (AEs) were calculated as
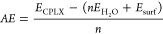
where *E*_CPLX_ is the energy of the complex (water adsorbed on the surface); *E*_H_2_O_ and *E*_surf_ are the energies of the isolated water molecule and the bare surface, each one calculated at its relaxed geometry; *n* is the number of adsorbed water molecules per surface unit cell. The transition state was localized with the DIMER method;^37,38^ as a starting structure, the proton was placed in the middle point between the O of the water and the Fe atoms of the product.

Vibrational frequencies were computed at the Γ point, by numerical differentiation of the analytical first derivatives, using the central difference formula (i.e., two displacements of 0.015 Å for each atom in each (*x*, *y*, *z*) direction), to confirm that the optimized structure is a minimum (all real frequencies) or a transition state (all real frequencies but one). To simulate IR spectra, the Phonopy^39^ code was used for both generating atomic displacements and processing VASP outputs. To plot the IR spectra, the convolution of intensities was done with Lorentzian functions and a fwhm (full width at half-maximum) of 50 cm^–1^. Thermochemistry was corrected using the quasi-harmonic approximation, proposed by Grimme,^40^ in which all frequencies below 100 cm^–1^ are replaced by free rotor modes. This improves the calculation of the thermal corrections, which would be otherwise underestimated using very low frequency values. To avoid discontinuity close to the cutoff, a damping function was used to interpolate the values computed within the two ranges of frequencies. To recover the systematic error due to the methodology and to the anharmonic nature of the O–H vibration, simulated O–H harmonic stretching frequencies were scaled for a proper factor calculated as
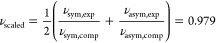
where ν_sym, exp_ and ν_asym, exp_ are the experimental symmetric and antisymmetric stretching of the isolated water molecule as taken from NIST, whereas ν_sym, comp_ and ν_asym, comp_ are the calculated ones in the harmonic approximation with the present methodology, on an optimized single water molecule in a unit cell of *a* = 20 Å, *b* = 20 Å, and *c* = 20 Å to ensure negligible lateral interactions among water replicas. The final scaled water monomer stretching frequencies are 3761 and 3652 cm^–1^ for the symmetric/antisymmetric modes. The bending mode at 1585 cm^–1^ was unscaled as the match of computed and experimental results is already good.

To simulate the phase diagrams, the reaction considered was the following:
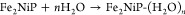
where Fe_2_NiP is the bare surface, *n*H_2_O is the number of water molecules in the gas phase, and Fe_2_NiP-(H_2_O)_*n*_ is either molecular or reacted water adsorption cases with a different number of water molecules, depending on the wetting degree. As for each species we have calculated the vibrational frequencies, we have also included the dependence on temperature and pressure of the water addition according to the following equation:^41^
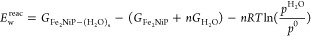
where *G* is the Gibbs free energies of each system, *R* is the ideal gas constant, *T* is the temperature, *p*^H_2_O^ is the water pressure, and *p*^0^ is the atmospheric pressure (assumed to be 1 atm).

Electric properties were calculated throughout the VASPKIT python package,^42^ whereas Bader’s charge analysis was performed with the code developed by the Henkelman group.^43−46^

The kinetic rate constant of water dissociation was computed using the Harmonic Transition State Theory:^47^
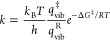
where *q*_vib_^R^ and *q*_vib_^‡^ are the vibrational partition functions for reactant and transition state, with the latter deprived of the loose vibrational mode that corresponds the motion leading to the reaction; *k*_B_ and *h* are the Boltzmann and Planck constants, *T* is the absolute temperature; *R* is the ideal gas constant; Δ*G*^‡^ is the difference of free energy between the transition state structure and the previous corresponding minimum. The half-life time *t*_1/2_ has been estimated assuming first-order kinetics as
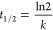


Visualization and manipulation of the structures and figure rendering were done with the MOLDRAW,^48^ VMD,^49^ and POVRAY^50^ programs.

## Results

### H_3_PO_3_ and H_3_PO_4_ Formation at the (110) Fe_2_NiP Surface

**Figure 1:**
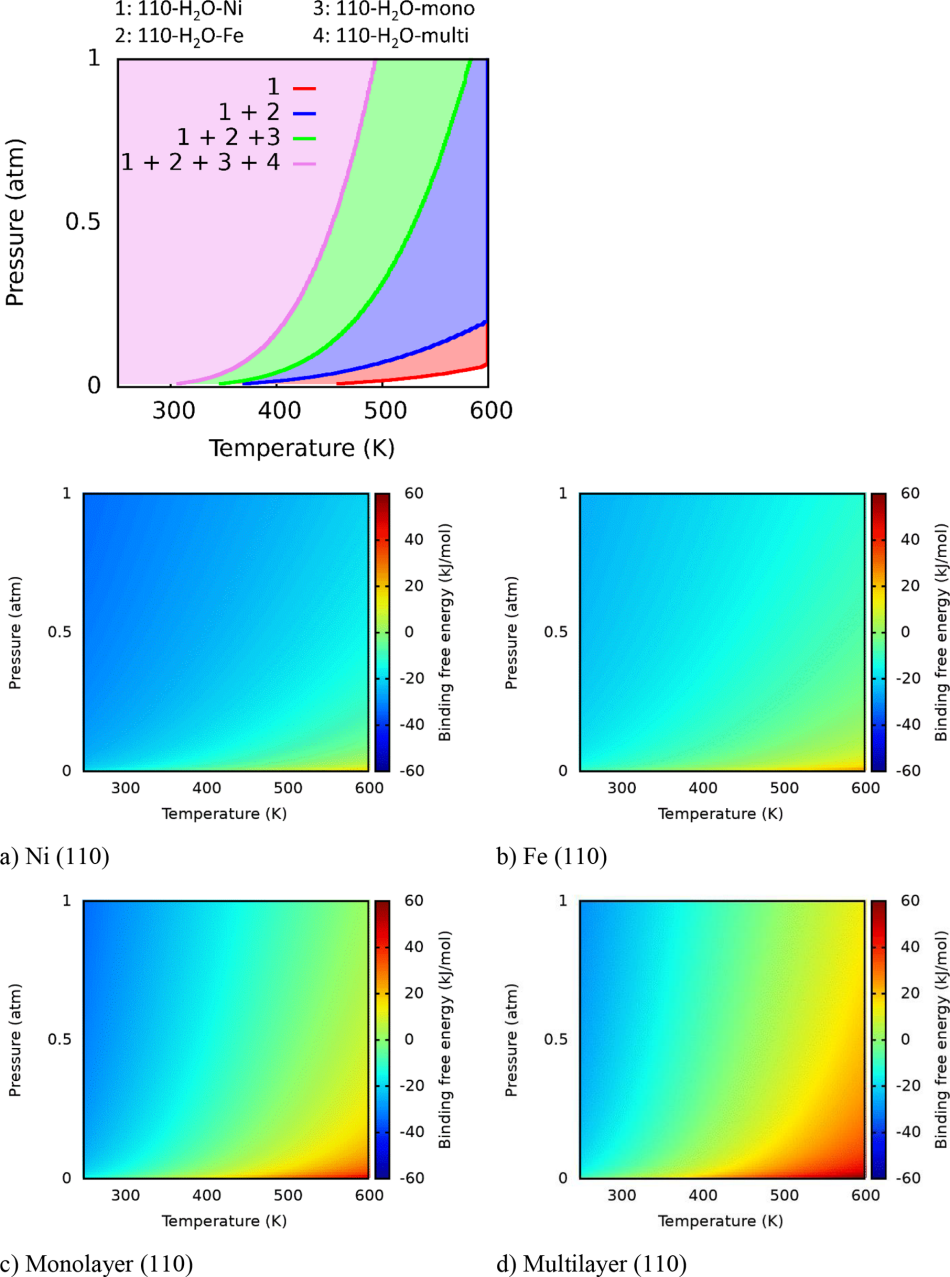
Phase diagram stability of water molecularly adsorbed on the schreibersite (110) surface as a function of temperature and water pressure: (a–d) individual adsorption free energy of single water (a and b), water monolayer (c), and water multilayer (d) cases as a function of temperature and water pressure.

In our recent paper, we demonstrated that water favorably interacts with the most stable (110) Fe_2_NiP surface. We briefly recall in a one-shot image the region of water adsorption stability from a single water molecule up to the multilayer depending on temperature and pressure: Figure 1a shows the phase diagram for all the cases reported in Figure 4 of ref (27). Each line corresponds to the boundary of stability between two structures, and the chart should be read from left to right, where at low temperature (left, pink color region), all systems are stable (the multilayer and, therefore, also the monolayer and the single water molecule adsorptions). Moving from the pink to the green region, the water multilayer becomes unstable, only leaving the physisorbed monolayer, and last, only the single water adsorptions (blue for Fe and red for Ni) remain stable. In Figure 1b–e, the binding free energy is reported as a colored plot for each separate system; the zero of the binding energy (green color) corresponds to the solid lines of Figure 1a.

**Figure 2:**
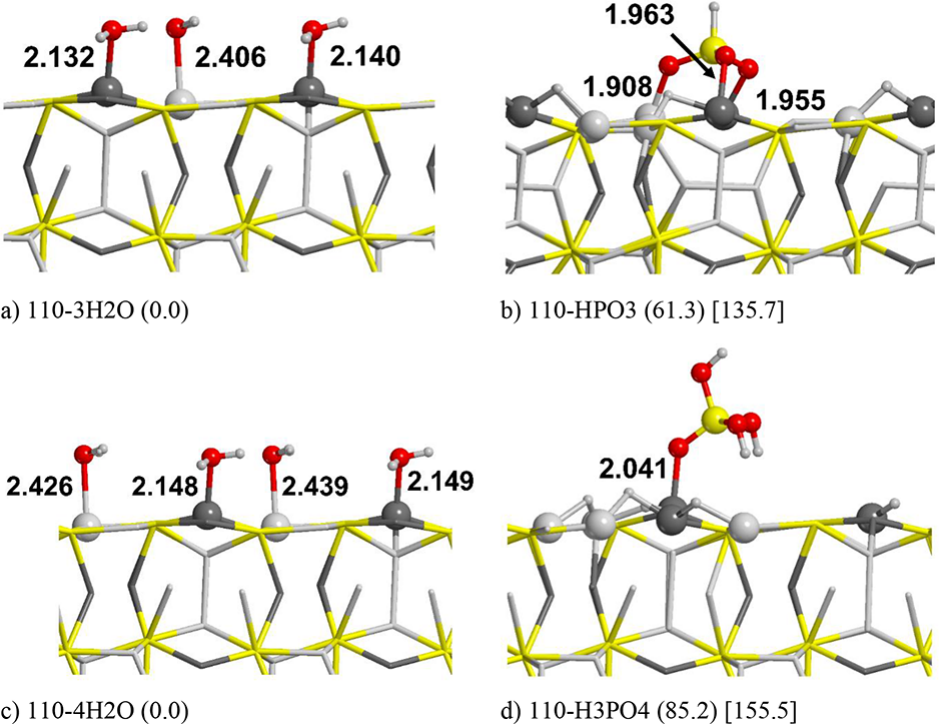
PBE-D*0 optimized structures of reactants (left column) and products (right column) of the phosphite (top) and phosphate (bottom) formation reactions on the (110) Fe_2_NiP surface. Energy values are in kJ/mol. Numbers in round parentheses correspond to energetic values in the gas phase; numbers in square parentheses, in PCM (water). Atom color legend: H in white, O in red, P in yellow, Fe in light gray, and Ni in dark gray.

In our previous paper, we also showed that water dissociation at the (110) surface, i.e., the first step of the pathway toward any phosphorus oxygenated compound formation, is an endergonic process. According to these results, the (110) Fe_2_NiP surface is not a good candidate to promote the corrosion process bringing to a hydroxylated/hydrogenated metallic surface. Nevertheless, we simulated the formation of the following products: phosphonic acid (H_3_PO_3_) and phosphoric acid (H_3_PO_4_) in their molecular and deprotonated forms (see Figure 2), each one starting from the corresponding reactants with three and four water molecules molecularly adsorbed on a (110) 2 × 2 supercell. To start from a chemically sensible structure of the considered products, one of the outermost P atoms was extracted from the main surface (see Figure S16 for details) by about 2 Å along the *z* (nonperiodic) axis and surrounded by H/O/OH species up to the formation of the desired product. The remaining H atoms (three in the case of H_3_PO_3_ and five in the case of H_3_PO_4_) were placed to saturate metallic atoms on the surface. The reactions were also simulated using the same species as products but considering their deprotonated partners using the corresponding H atoms to bind other metallic sites on the surface. As one can see for both products, the reactions are endergonic: Δ*E* = +61.3 and +85.2 kJ/mol for 110-HPO3 and 110-H3PO4, respectively. The structures were also optimized in continuous water solvent, and in these cases, the endergonicity increases compared to the solvent-free reactions: Δ*E* = +135.7 and +155.5 kJ/mol for 110-HPO3 and 110-H3PO4, respectively. Curiously, the most stable form of the phosphonic acid is the deprotonated one (HPO_3_^2–^), in contrast to the molecular one for the phosphoric (H_3_PO_4_) acid. Figure S11 shows the electric field and work function of water physisorption models outside the (110) surface, whereas Figure S12 shows the cases for dissociative adsorption. In contrast to what happens on systems like RuO_2_, which promote water dissociation,^51^ here the water coverage increases the work function values, indicating that water reactivity is hindered on this surface, in agreement with the higher energy of the product (and also with their corresponding work functions). This is no longer true for the water monolayer case, even if we believe that this can be an artifact of the PBE, for the critical description of both the covalent (O–H) and noncovalent (O···H) interactions in water, as we already discussed in our recent paper.^27^ Indeed, in that structure, an incipient proton transfer occurs, making the extraction of an electron easier, thus giving a lower value of the work function. The electric field experienced by the outermost part of the surface (the peaks at 9–10 and 30–31 Å) decreases from 24.75 V/Å for the bare surface to 20.12 V/Å for the water multilayer, following more or less the inverse of the adsorption energy values; i.e., water produces a shielding effect to the surface by minimizing its electric field when the surface is at water saturation. In the case of water dissociation (Figure S12), the work function increases with respect to molecular adsorptions, thus indicating that, on this surface, charge transfer processes are difficult to occur and, accordingly, also the water dissociation. Finally, Table S1 shows the results of atomic spin and charges of the bare surfaces and gas phase water molecule (as reference) and water chemisorption cases. In the case of water physisorption, no relevant charge transfer processes are observed (see ref (27)), at variance with the not negligible surface charge transfers for chemisorption. The most affected atoms are H and P, with a charge difference with respect to the reference systems (water for H and the bare schreibersite surface for P) of about −0.8/–0.9 and +1/+4 *e*, respectively. Obviously, the P element exhibits more possibilities in terms of oxidation numbers depending on the neighboring atoms (Table S1). This means that charge transfer processes are fundamental to activate the corrosion process, and this piece of information, joined to that given by the work function, provides a clear answer on the feasibility of this process that on the most stable (110) surface is not favorable.

### H_3_PO_3_ and H_3_PO_4_ Formation on the (001) Fe_2_NiP Surface

The less stable (001) Fe_2_NiP surface is a minor but still relatively important fraction of a generic schreibersite nanoparticle, as shown by the Wulff construction (as described in full details in ref (26)); i.e., two out of six faces of the parallelepiped shown in Figure S14 are due to the less stable (001) surface. This fraction, however, changes depending on the temperature, and in particular, higher temperatures tend to favor the (001) surface over the (110). The Wulff constructions reported in Figure S14 are modeled using the surface free energies in a wide range of temperatures from 0 to 3000 K. The percentages of contribution to the total surface area are 64.9 for (110) and 35.1 for (001) at 0 K (only energy + ZPE), 64.7 for (110) and 35.3 for (001) at 125 K, 64.3 for (110) and 35.7 for (001) at 298 K, 63.5 for (110) and 36.5 for (001) at 600 K, and 56.4 for (110) and 43.6 for (001) at 3000 K. The latter was chosen to take into account the possibility of a surface reconstruction driven by the extreme postshock temperatures due to a meteor impact, which can easily reach >2000 K.^52−54^

**Figure 3:**
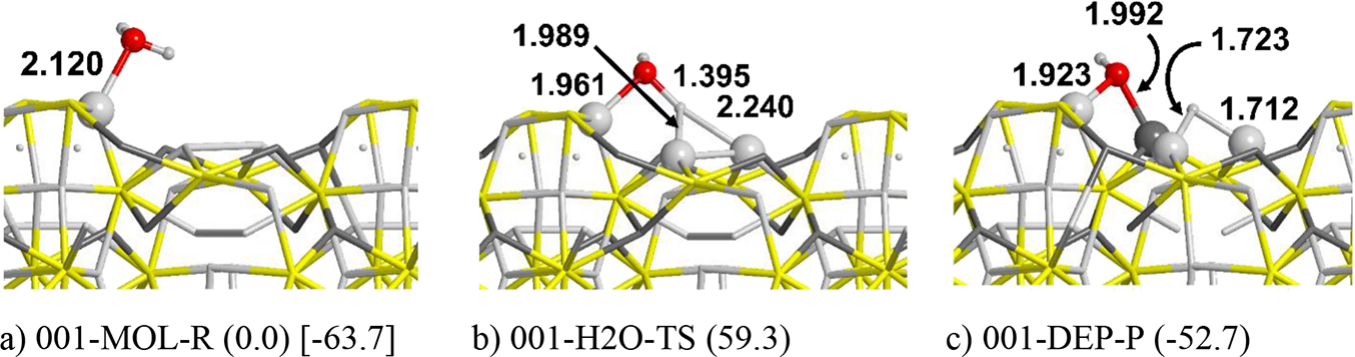
PBE-D*0 optimized structures of the water dissociation process on the (001) Fe_2_NiP surface. Energy values in kJ/mol. In round parentheses: the relative energy taking the free reactants as reference. In square parentheses: the adsorption energy relative to the free reactants. Atom color legend: H in white, O in red, P in yellow, Fe in light gray, and Ni in dark gray.

Figure 3 shows the dissociation of an isolated water molecule adsorbed on the (001) surface. The higher reactivity of the (001) surface with respect to (110) is already clear from the molecular water adsorption energy, which is almost twice for (001) (AE = −63.7 kJ/mol) compared to (110) (AE = −37.6 kJ/mol). Furthermore, the dissociative adsorption is, in this case, exergonic (Δ*E* = −52.7 kJ/mol), in contrast to the (110) surface (Δ*E* = 29.1 kJ/mol, see Figure S1). We also calculated the dissociation kinetic barrier (Figure 3b, Δ*E* = 59.3 kJ/mol), which gives a half-life time of 6.3·10^–6^ h (0.02–0.03 s, basically instantaneous) for water dissociation at 298 K, which increases to 3.5·10^9^ h (10^5^ years) at the low temperature of 125 K (see Figure S2 for further details). Although in the present work only the very first step of the corrosion process was simulated (i.e., water deprotonation), these kinetic results somehow match with the experiments performed in refs. (13) and (17) where, at low temperature, the IR bands does not show any other signal apart that of water,^13,17^ while at room temperature, the reaction appears to be instantaneous, as confirmed by the new feature at 2423 cm^–1^.^13,17^ According to our results on Bader’s charges (Table S1), we can say that the bond breaks homolitically, as revealed by a total charge of 0.923 *e* in the 001-H2O-TS (the H atom keeps its own electron), further increasing in the product due to electron flux from the surface toward the more electron negative H compared to Fe and Ni atoms. The charge difference between the O atom of the OH moiety and that of H_2_O is almost negligible (+0.076 and +0.032 for 001-H2O-TS and 001-DEP-P, respectively), as it still keeps its original charge due to an electron flux coming from the metallic surface counterbalancing the one electron lost from the OH group. This means that, when water interacts with the surface through “simple” physisorption, the charge transfer between the adsorbate and the surface is very weak, but when water dissociates (i.e., chemisorption), a redox reaction occurs; i.e., the corrosion process is taking place.

**Figure 4:**
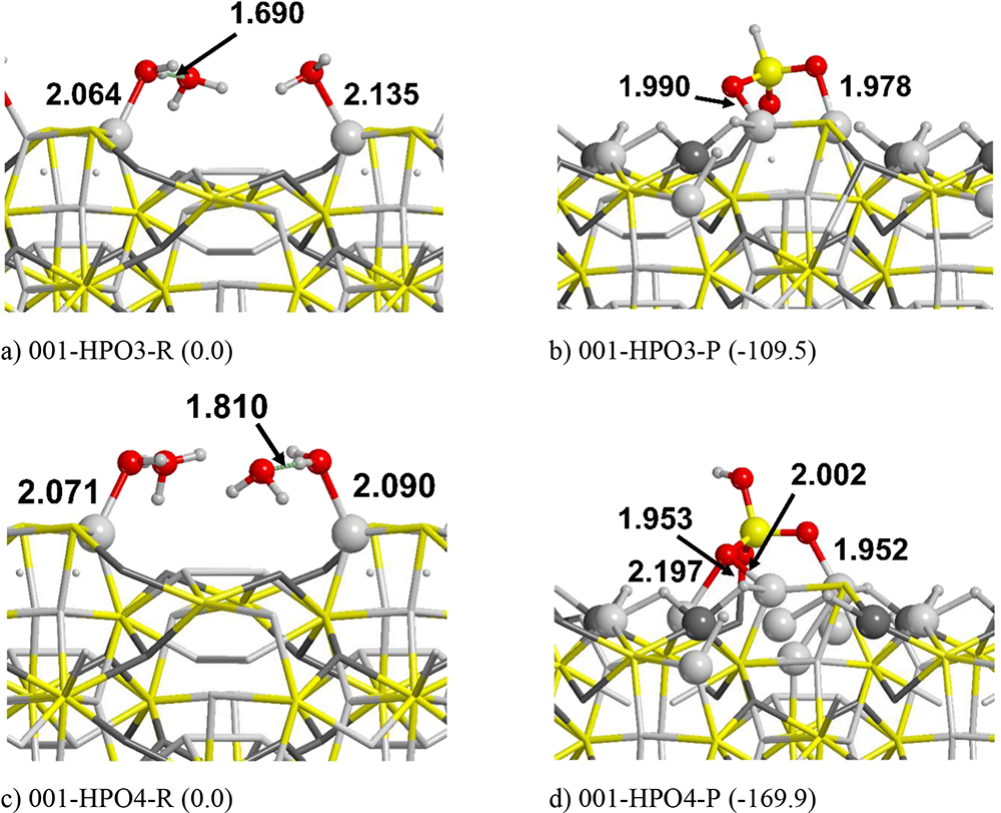
PBE-D*0 optimized structures of reactants (left column) and products (right column) of the phosphite (top) and phosphate (bottom) formation reactions on the (001) Fe_2_NiP surface. Energy values are in kJ/mol, and they refer to the “on surface” reactions. Atom color legend: H in white, O in red, P in yellow, Fe in light gray, and Ni in dark gray.

In view of these results, we simulated the reactions of phosphite and phosphate formation (as on the (110) surface). The results are summarized in Table 1, and the optimized structures are shown in Figure 4. Because of the higher reactivity of the (001) surface compared to the (110) one, we simulated the products in their deprotonated state to maximize the number of anchoring points with the metal atoms of the surface. It turns out that, in most of the cases, the reaction is strongly exergonic (around −200/–250 kJ/mol), and accordingly, we investigated the reactivity of this surface in more detail. In Figure 4, the “on surface” reactions refer to all the processes occurring at the surface; i.e. the energies are taken from the complexes (001-HPO3-R and 001-HPO4-R as reference for the reactants and 001-HPO3-P and 001-HPO4-P as products); in contrast, “gas to gas” refers to the values calculated with the adsorbates far from the surface (i.e., (001) + three to four gas-phase H_2_O as reference for the reactants and (001) + gas-phase H_3_PO_3_–H_3_PO_4_ as products). This means that, in the “gas to gas” processes, the reactants are the bare Fe_2_NiP surface and the isolated water molecule, taken *n* times to ensure the mass balance, whereas the products are the isolated H_3_PO_3_ and H_3_PO_4_ molecules and the hydrogenated surfaces (three and five H atoms, respectively, for the phosphite and phosphate formation reactions). The most important difference between the “on surface” and “gas to gas” simulations is in the entropic difference between reactants and products, as the number of species with translational degrees of freedom is different for the two cases. Depending on the reference and the conditions (gas phase or PCM), in one case, the H_3_PO_3_ formation is slightly endergonic (Δ*E* = 11.1 kJ/mol), but for the H_3_PO_4_, in all cases, the reaction is strongly exergonic (see Table 1). The simulations were also carried out in PCM, which tends to lower the reaction energy. We want to highlight that, in the case of “gas to gas” reactions simulated in PCM, the rotational and translational contributions to the entropy were removed in the free energy correction, as in liquids, molecular rotations and translations are partially hindered.

**Table 1:** Reaction Energies (Δ*E*'s), Enthalpies (Δ*Hs*), and Gibbs Free Energies (Δ*Gs*) of the HPO_3_^2–^ and HPO_4_^2–^ Formation Reactions.^a^

	gas phase			PCM (water)		
001-HPO3-P						
reaction type	Δ*E*	Δ*H* [298]	Δ*G* [298]	Δ*E*	Δ*H* [298]	Δ*G* [298]
on surface	–109.5	–166.3	–151.9	–56.6	–117.0	–101.3
gas to gas	–13.9	–14.4	–55.9	11.1	–5.5	–58.9
001-HPO4-P						
reaction type	Δ*E*	Δ*H* [298]	Δ*G* [298]	Δ*E*	Δ*H* [298]	Δ*G* [298]
on surface	–169.6	–252.1	–227.7	–139.1	–209.5	–193.6
gas to gas	–168.4	–175.9	–228.3	–129.6	–158.6	–204.6

a In parentheses, the temperature at which the thermodynamic corrections were calculated. Values are in kJ/mol.

Finally, as the water corrosion reaction of Fe_2_NiP also produces H_2_ as byproduct,^12,15^ we simulated the physisorption of H_2_ molecules, starting from the 001-HPO3-P and 001-HPO4-P cases and using the chemisorbed H atoms to form 2 and 3 H_2_ molecules, respectively (see Figure S5). Both reactions become endergonic (Δ*E*^001-HPO3-H2^ = 57.9 and Δ*E*^001-HPO4-H2^ = 56.4 kJ/mol), indicating that the H_2_ production is not favorable, as its formation probably requires a complete saturation of all sites by the chemisorbed H atoms. Another possibility is that the production of H_2_ is due to redox reactions occurring among intermediates of oxidized phosphorus and with H_2_O molecules, without a direct participation of the surface to the reaction, which is, however, not studied here.

### Schreibersite Mineral toward Phosphates: the Jungle of a Reaction Pathway

**Figure 5:**
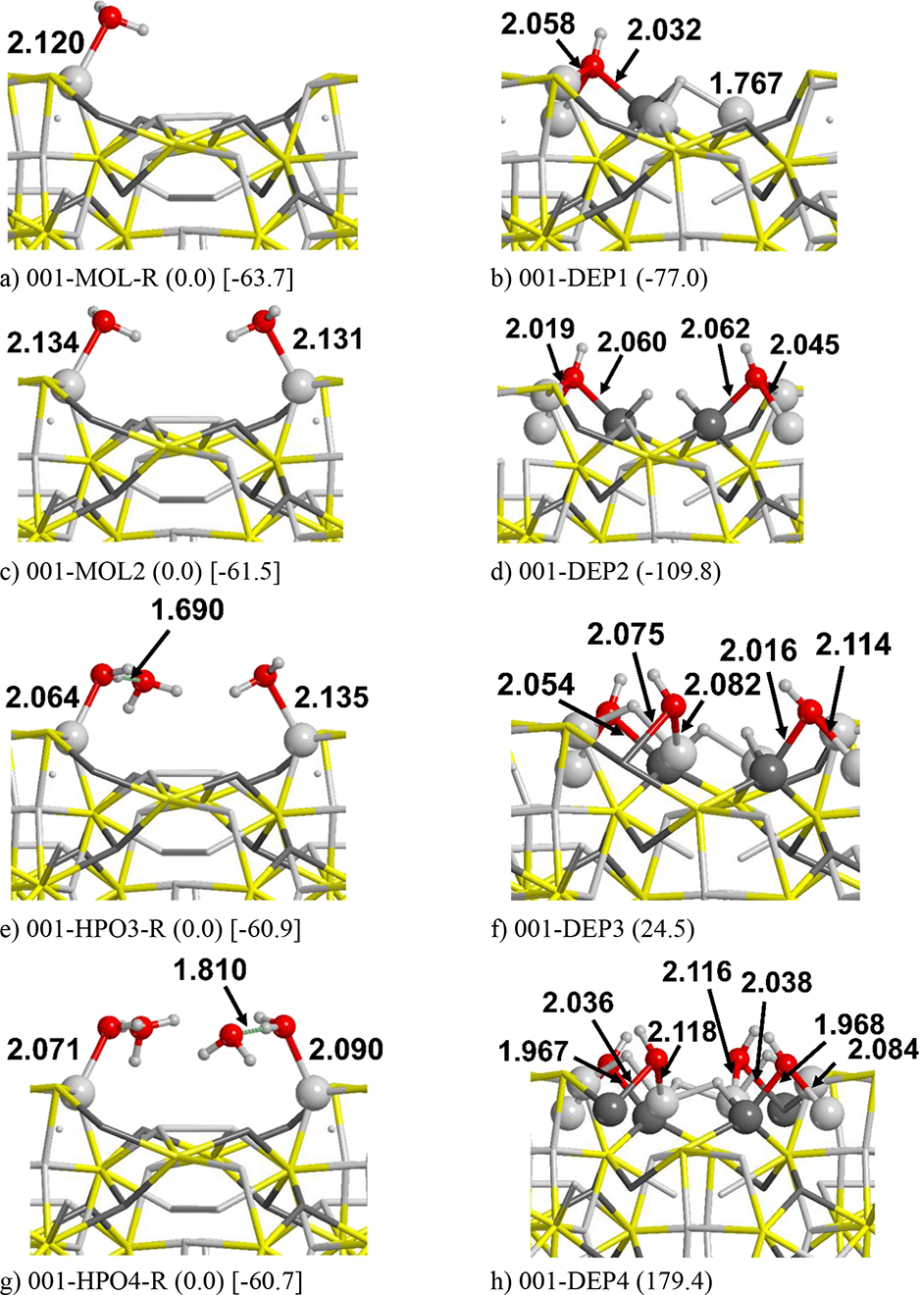
PBE-D*0 optimized structures of successive water dissociation on the (001) Fe_2_NiP surface, from one to four molecules, according to the following reaction: H_2_O → H + OH. Energy values in kJ/mol. In round parentheses: the dissociation energies using the molecular adsorption as reference. In square parentheses: the adsorption energy. Atom color legend: H in white, O in red, P in yellow, Fe in light gray, and Ni in dark gray.

**Figure 6:**
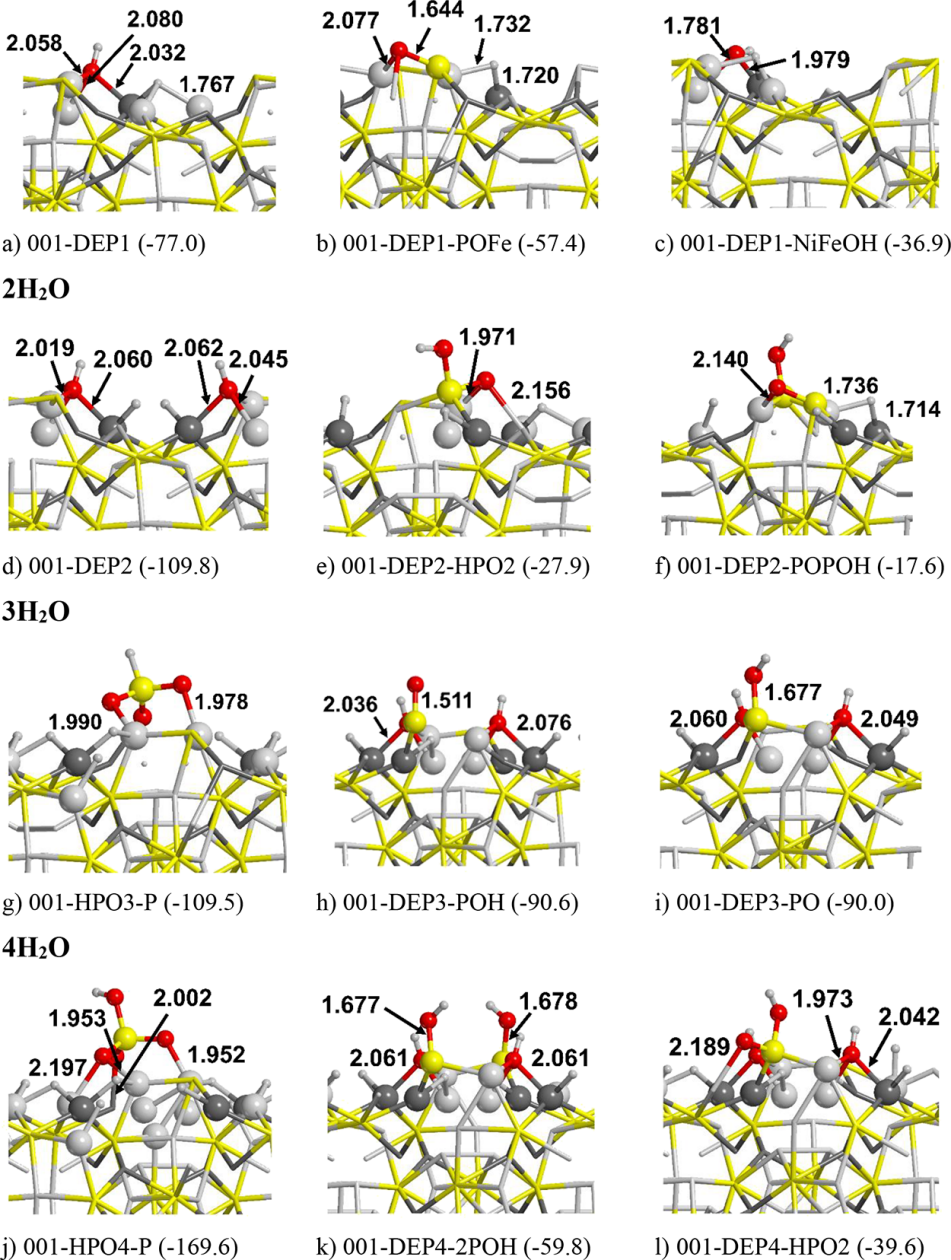
PBE-D*0 optimized structures of the most stable deprotonated structures (from one to four H_2_O molecules) on the (001) Fe_2_NiP surface. Energy values in kJ/mol. Atom color legend: H in white, O in red, P in yellow, Fe in light gray, and Ni in dark gray.

The reaction pathway, which accounts for the global wetting process of schreibersite, from the adsorption of water molecules (physisorption) and their dissociation (chemisorption) to the finally oxygenated phosphorus compounds, is very complex. Other pathways could dominate over the formation of phosphorus oxygenated compounds: for example, if water preferred to bind exclusively to metal atoms than phosphorus, all of the possible scenarios should therefore be taken into account. Figures 5 and 6 show several adsorptions of water in both its molecular and dissociated states, also increasing the number of water molecules from one to four (the minimum number to obtain H_3_PO_4_). In Figure 5a,b, the dissociation of one H_2_O atom is shown. As one can see, molecular water adsorbs preferentially on a single metallic site (in this case Fe, see Figure S3a) rather than sharing different bonds with more than one metallic atom (see Figure S3d, 001-MOL-FeFe), whereas their OH and H fragments are shared between several metal atoms, thus contributing to stabilize the dissociated form compared to the molecular one (see Figure 5b,d,f,h). Figure 5b represents the most stable way to dissociate a single water molecule on this surface (Δ*E* = −77.0 kJ/mol). As a consequence, we added other water molecules and their corresponding dissociated forms following the same “dissociating strategy” to see if successive water dissociations on metal atoms represent a more favorable pathway than dissociation on phosphorus. The dissociation of 2H_2_O (Figure 5c,d) leads to an even more stable product (Δ*E* = −109.8 kJ/mol, not normalized per water molecule). Despite that, the stabilization does not increase linearly with the number of water molecules as the Δ*E* value does not double with respect to the single H_2_O dissociation. The addition of other water molecules, indeed, leads to a stability inversion with respect to the molecular form: Δ*E* = 24.5 and 179.4 kJ/mol for the dissociation of 3 and 4H_2_O, respectively; i.e., the addition of other water molecules stabilizes the molecular adsorption of water rather than the dissociated ones. With these few data, we can speculate that water dissociation exclusively occurring on metal atoms is not a favorable thermodynamic process. Indeed, at a certain degree of water coverage, the attack of water on phosphorus becomes energetically more convenient. Figure 6 shows a sampling of the most stable structures with 1 to 4H_2_O molecules. With 1 and 2H_2_O, we found the same number of stable structures as those for Figure 5 (001-DEP1, 001-DEP2). In the structures ordered along decreasing stability, we found many cases where the O atom or the OH moiety binds covalently with phosphorus (forming P=O and P–OH moieties, Figure 6b,e,f). These forms are less stable than 001-DEP1 and 001-DEP2 but still show a negative Δ*E* with respect to the H_2_O molecularly adsorbed on either Fe or Ni atoms. Figure 5 shows that with 3H_2_O, the saturation of other metal atoms with H and OH atoms is no longer an energetically favorable process. Therefore, we focused our attention to the phosphorus. From Figure 6h,i where P–OH and P=O moieties are formed, it is clear that our initial hypothesis is confirmed: the saturation of phosphorus at a certain point becomes a favorable process, with reaction energies of Δ*E* = −90.6 and −90.0 kJ/mol for 001-DEP3-POH and 001-DEP3-PO, respectively, very close to the formation of the phosphite (001-HPO3-P, Δ*E* = −109.5 kJ/mol). Similarly, for the 4H_2_O cases, the water dissociating on phosphorus led to stable structures, the most stable being 001-HPO4-P (phosphate ion). In conclusion, the (001) surface of Fe_2_NiP allows the corrosion process to be operated by water adsorption, at least from a thermodynamic point of view (and also from a kinetic point of view limited to the dissociation of a single H_2_O molecule). The corrosion process starts from the metal atoms (Fe and Ni) to finally involve, on subsequent steps, also phosphorus, possibly leading to its oxygenated forms, keys for the life processes.

Figure S15 shows the phase diagram including all the studied models of Figures 4–6 and Figure S4, thus also including the water monolayer (001-MONO) adsorption and its corresponding dissociated form (001-MONO–DEP). The thermodynamic corrections completely change the stability scenario given by the sole electronic energy considerations. Indeed, the majority of the structures are not present in the phase diagram: at very low temperatures (below 100 K), the water monolayer represents the most stable structure at every water pressure. At higher temperatures, almost all the chart is occupied by 001-HPO4-P, i.e., the phosphate, and this is one of the most interesting results of the present work if we consider the high temperatures meteorites can reach, either in the deep space and especially when they land on a planet.

Finally, Figure S13 shows the electric field and work function of water physisorption and chemisorption models on the (001) surface. The main difference with respect to the (110) surface is the work function of all the H_2_O-bearing models, which is lower with respect to that of the bare surface; this indicates that, on the (001) surface, the charge transfer processes and, accordingly, the water reactivity are enhanced with respect to the (110) one. In this case, we highlighted two peaks (one more exposed to the vacuum and one representing a more internal part of the surface) because the (001) surface presents a much more corrugated shape with respect to the flat (110). As a general trend, as already reported for the (110) surface, the two peaks of the electric field produced by the surface decrease their values with the increasing water saturation despite the inversion for some cases. This happens in those cases where OH^–^ moieties, which bring a net negative charge, are present in the outermost part of the surface. Indeed, in the progression from 001-DEP1 to 001-DEP4, the electric field increases almost linearly: 8.08 V/Å (001-bare), 9.35 V/Å (001-DEP1), 10.41 V/Å (001-DEP2), 10.91 V/Å (001-DEP3), and 12.06 V/Å (001-DEP4).

### Schreibersite (001) IR Spectra

In Figures S6 and S7, we reported the simulated IR spectra of either physisorption or chemisorption cases on the (001) surface. For the cases of H_2_O molecularly adsorbed on Fe_2_NiP (Figure S6a,c,e,g), the position of the peaks (stretching at 3000–3500 cm^–1^ and bending at around 1600 cm^–1^) and, in some cases, also the relative intensities are well reproduced when comparing with the experimental one.^13^ We are reminded, however, of an open question from our previous work regarding the IR spectra,^27^ which deals with the experimental peak at 2423 cm^–1^ not reproduced at all by our simulations. It was originally ascribed to water chemisorption and in particular to an incipient deprotonation and formation of a H_3_O^+^/OH^–^ ion pair. In ref (17), the peak at 2423 cm^–1^ was attributed to the formation of P–H but without specifying the phosphorus oxidation state. On the (110) surface, we already demonstrated that the simple P–H group in the chemical environment of Fe_2_NiP cannot account for that feature as it vibrates at 2204 cm^–1^, i.e., 200 cm^–1^ bathochromically shifted from the expected result. This difference is going to become larger when anharmonicity is taken into account, as the absolute wavenumber of P–H is expected to decrease. A similar case was also simulated at the (001) surface (one of the less stable (Δ*E* = 16.1 kJ/mol) surfaces), as well as the corresponding IR spectrum, reported in Figure S7c. The P–H in this case vibrates at 2194 cm^–1^, which confirms the results obtained on the (110) surface, thus possibly excluding this particular moiety from the possible contributions to the experimental peak at 2423 cm^–1^. Our hypothesis is that that peak is indeed due to a P–H stretching mode but in an advanced reaction stage with water, i.e., when the phosphite moiety is formed. The phosphate cannot be included as a possible candidate to disentangle this problem because in that case only P–O bonds are present, which vibrate at lower wavenumbers (ca. 1000 cm^–1^).

Therefore, we calculated the IR spectra of several different P oxygenated compounds in the gas phase containing a P–H moiety, and the results are summarized in Figure S8. We adopted the Gaussian code to perform high-level calculations (using the B2PLYPD3 double hybrid functional to be compared with PBE-D*0) and also to rigorously introduce anharmonic effects. Table S2 clearly shows that there is a systematic difference between B2PLYPD3 and PBE-D*0 results. However, the anharmonic results obtained at the B2PLYPD3 level match very well with the harmonic results provided by PBE-D*0. This is due to the fact that PBE-D*0 underestimates the force constant of X–H bonds (we already discussed in our previous paper this effect applied to O–H bonds in water),^55^ but because of a fortuitous compensation error, the underestimation is of the same order of magnitude of the anharmonicity (at least for these specific P–H cases). Therefore, we can trust the PBE-D*0 results without applying any correction to the harmonic frequencies (as we did instead for the O–H bonds). We want, however, to further highlight that this is not a general behavior but a workaround that in this specific case was carefully checked. Moreover, from Table S2, a general trend can be observed: the more deprotonated the species is, the higher is the bathochromic shift of the P–H bond. This is due to the fact that deprotonating an OH moiety produces a larger separation of charge between P and the other atoms; i.e., whereas O easily handles negative charges by shortening the P–O bonds due to deprotonation, the P–H bond, on the contrary, becomes longer (see Figure S9). In water, this effect is less pronounced, as the negative charges on O atoms are partially mitigated by the solvent. On the Fe_2_NiP surface, we have already seen that the O atoms directly interact with the outermost metal atoms, and accordingly, the distribution of charge is similar to that of H_3_PO_3_. Indeed, in Figure S6b,d, where the IR spectra of HPO_3_^2–^ adsorbed on the (001) surface are shown, both in gas phase and in PCM, the P–H vibrates at 2437 and 2467 cm^–1^, respectively, very close to the values of the isolated H_3_PO_3_ molecule (2443 and 2454 cm^–1^ in gas phase and in PCM, respectively). In Figure S10, we reported the correlation between P–H distance and the wavenumber at which this moiety vibrates; as one can see, the correlation is excellent even for very small bond changes, with the frequency being a very sensitive property with respect to any structural change. The difference between the two figures is that in Figure S10b, the DFT result on the (001) surface is added to Gaussian molecular calculation; even if the comparison is between different programs that treat the atomic orbitals in a very different way (plane waves vs localized functions), the correlation is very good, and it also improves the *R*^2^ of the fitted line. After these careful checks to prove the goodness of PBE-D0* results on the P–H frequencies, we can say with good confidence that the elusive peak at 2423 cm^–1^ belongs to the H_3_PO_3_ molecule (2443/2454 cm^–1^, in the gas phase and in PCM) or to a H_*x*_PO_3_^(3–*x*)^−^^ group (HPO_3_^2–^ in the specific case, 2437/2467 cm^–1^ in the gas phase and in PCM) adsorbed on the schreibersite surface.

## Conclusions

In the present work, the corrosion process of schreibersite operated by water was studied by means of periodic simulations carried out within the DFT framework, inclusive of the dispersion contribution adopting static calculations.

**Figure 7:**
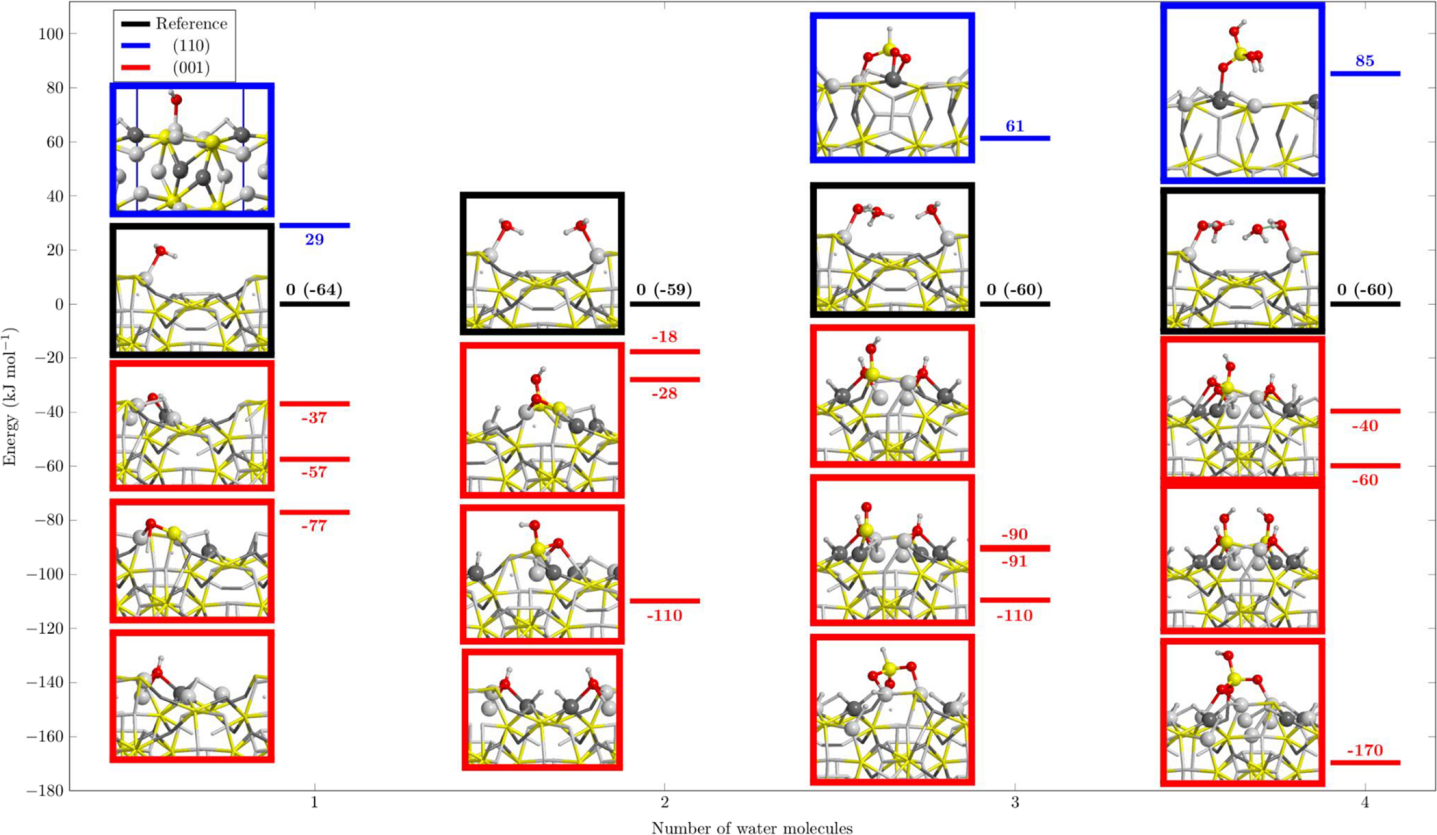
PBE-D*0 optimized structures of the most stable deprotonated structures (from one to four H_2_O molecules) on the (110) (above the reference) and (001) (below the reference) Fe_2_NiP surfaces (the black line as a reference corresponds to the molecular adsorptions, in parentheses: the adsorption energy due to addition of a H_2_O molecule). Energy values in kJ/mol. Atom color legend: H in white, O in red, P in yellow, Fe in light gray, and Ni in dark gray.

Figure 7 reports a graphical summary with all of the most stable H_2_O–Fe_2_NiP surface reactions on both (110) and (001) surfaces. Black lines correspond to the reference for both the surfaces, on which the water molecules (from one to four) are physisorbed. All the reactions of water on the (110) surface lead to unstable products, highlighted with the blue lines, whereas the corrosion products on the (001) are well below the black lines. In particular, for the (001) surface, it is interesting to note that the most stable products of the reactions with 3 and 4H_2_O molecules are the phosphite (HPO_3_^2–^) and the phosphate (HPO_4_^3–^). Moreover, we also demonstrated, with a good level of confidence, that the peak at 2423 cm^–1^ in ref (13) that arises at high temperatures (298 K) and is not present at low temperatures (125 K),^17^ is due to the formation of the H_3_PO_3_ molecule (2443/2454 cm^–1^, in gas phase and in PCM) and/or to an adsorbed phosphite (2437/2467 cm^–1^ in gas phase and in PCM), and accordingly, it is particularly useful to follow the water corrosion process because it is in a “clean” zone of the IR spectrum. On the contrary, the phosphate species only present P–O and O–H moieties that vibrate at 1000 and 3500 cm^–1^, respectively, and that can be therefore hidden by other species.

We did not explore the complexity of the kinetic reactions in this work; nevertheless, the favorable thermodynamic results related to water adsorption on the less stable (001) surface give credit to the potential role of schreibersite as the source of biogenic phosphorus. This system certainly deserves to be further studied to fully characterize the entire potential energy surface (inclusive of transition state structures), also considering other potential reactive channels not accounted for in the present work, bringing other phosphorus oxygenated compounds. Furthermore, the study can be extended toward the reactivity of nucleotides and sugars in conjunction with that of water. It is possible that the energy released by the formation of surface phosphates is the key to promote, in turn, the reaction to integrate the newly generated phosphates into the aforementioned building blocks of life.

## Supporting Information Available

Bader charge analysis, frequencies of H_3_PO_3_ and deprotonated forms, rate constant, additional structures, IR spectra, electric field of bare and corroded surfaces, temperature-dependent Wulff construction, and phase diagram of the most stable structures as a function of temperature and water pressure (PDF)
