# Supplementary material for: Computational Study on the Water Corrosion Process at Schreibersite (Fe2NiP) Surfaces: from Phosphide to Phosphates
Source: ACS Earth Space Chem. 2023 Sep 21;7(10):2050–61. doi: 10.1021/acsearthspacechem.3c00167 (PMC10591503; doi:10.1021/acsearthspacechem.3c00167)
Supplement: Supplementary file 1 — sp3c00167_si_001.pdf [file sp3c00167_si_001.pdf]

Computational Study on the Water Corrosion Process at Schreibersite (Fe_2NiP) Surfaces: from Phosphide to Phosphates

Supporting Information

Stefano Pantaleone,[*1,2] Marta Corno,[1] Albert Rimola,[3] Nadia Balucani,[2,4,5] and Piero Ugliengo*[*1]*

[1]Dipartimento di Chimica and Nanostructured Interfaces and Surfaces (NIS) Centre, Università degli Studi di Torino, via P. Giuria 7, I-10125, Torino, Italy

[2]Dipartimento di Chimica, Biologia e Biotecnologie, Università degli Studi di Perugia, Via Elce di Sotto 8, I-06123 Perugia, Italy

[3]Departament de Química, Universitat Autònoma de Barcelona, 08193 Bellaterra, Catalonia, Spain

[4]Osservatorio Astrofisico di Arcetri, Largo E. Fermi 5, I-50125 Firenze, Italy

[5]Université Grenoble Alpes, CNRS, Institut de Planétologie et d'Astrophysique de Grenoble (IPAG), F-38000 Grenoble, France

*E-mail: piero.ugliengo@unito.it, stefano.pantaleone@unito.it

Keywords: Meteorites, phosphorous problem, DFT, prebiotic chemistry, water adsorption

Computational details

Molecular simulations

All molecular calculations on gas phase isolated P oxygenated compounds have been carried with the Gaussian16 code. In order to obtain the most stable structures among the possible conformers the crest program was used at GFN2 level both for molecular and deprotonated structures. All conformers were re-optimized at B2PLYPD3/aug-cc-pvtz level and on the most stable structures vibrational frequencies were calculated. Geometry optimizations and frequency calculations were carried out also at PBE-D2/def2-QZVPP (see Figure S7) in order to obtain results to be compared with the VASP level of theory. Moreover, we also calculated the anharmonic corrections with both methods (Table S2) only for H_3PO_3 and its derivatives. It is well known that PBE underestimates the bond strength, and, accordingly, the same occurs to vibrational frequencies; curiously, the anharmonic frequencies at B2PLYPD3 level are very close to the harmonic ones at PBE level, at least for the description of the P—H bond. As it often happens in many approximated DFT functionals, thanks to the error compensation, experimental results and/or higher-level calculations are correctly reproduced. Of course, it is impossible to base results and a-priori conclusions on this behaviour of DFT, however we clearly demonstrate that we can trust on PBE harmonic frequencies at least to disentangle the problem of the peak at 2434 cm^{-1} (which is thought to belong to P—H stretching).

Table S1: Bader charge analysis of all the studied systems and total spin. Net charge and diff charge are calculated as difference between charges of the actual system with respect to the corresponding bare system. In the Atom column in parenthesis the moiety the principal atom belongs to. Units are in electron charge. The numbers in parenthesis correspond to the P charge with respect to gas phase H_3PO_3 and H_3PO_4.

H_2O gas

Atom	charge	net charge	diff charge	spin
O	7.168	-1.168	--	--
H	0.416	0.584	--	--

H_3PO_3 gas

Atom	charge	net charge	diff charge	spin
P	1.697	3.303	--	--
O (PO)	7.435	-1.435	--	--
H (PH)	1.412	-0.412	--	--
O1 (OH)	7.361	-1.361	--	--
H1 (OH)	0.366	0.634	--	--
O2 (OH)	7.364	-1.364	--	--
H2 (OH)	0.364	0.636	--	--

H_3PO_4 gas

Atom	charge	net charge	diff charge	spin
P	1.407	3.593	--	--
O (PO)	7.432	-1.432	--	--
O1 (OH)	7.330	-1.330	--	--
H1 (OH)	0.391	0.609	--	--
O2 (OH)	7.334	-1.334	--	--
H2 (OH)	0.387	0.613	--	--
O3 (OH)	7.350	-1.350	--	--
H3 (OH)	0.369	0.631	--	--

(110)

001-DEP4

Atom	charge	net charge	diff charge	spin
O	7.185	-1.185	-0.017	0.047
H (OH)	0.377	0.623	0.039	0.001
H	1.240	-0.240	-0.824	-0.017
O	7.186	-1.186	-0.018	0.048
H (OH)	0.377	0.623	0.039	0.001
H	1.240	-0.240	-0.824	-0.017
O	7.119	-1.119	0.049	0.067
H (OH)	0.415	0.585	0.001	0.004
H	1.252	-0.252	-0.836	-0.024
O	7.120	-1.120	0.048	0.067
H (OH)	0.414	0.586	0.002	0.004
H	1.253	-0.253	-0.837	-0.024

001-DEP1-POFe

Atom	charge	net charge	diff charge	spin
P	5.539	-0.539	0.032	-0.063
O (PO)	7.228	-1.228	-0.060	0.045
H1	0.381	0.619	0.035	-0.011
H2	1.346	-0.346	-0.930	-0.014

001-DEP2-HPO2

Atom	charge	net charge	diff charge	spin
P	3.717	1.283	1.855	-0.032

Atom	charge	net charge	diff charge	spin
P1	5.581	-0.581	--	--
P2	5.573	-0.573	--	--

(001)

Atom	charge	net charge	diff charge	spin
P1	5.572	-0.572	--	--

110-HPO3

Atom	charge	net charge	diff charge	spin
P	1.675	3.325	3.902 (-0.977)	0.008
H (PH)	1.414	-0.414	-0.998	0.002
O1	7.364	-1.364	-0.196	0.049
O2	7.375	-1.375	-0.207	0.029
O3	7.394	-1.394	-0.226	0.067
H1	1.210	-0.210	-0.794	-0.011
H2	1.207	-0.207	-0.791	-0.011
H3	1.211	-0.211	-0.795	-0.017
H4	1.230	-0.230	-0.814	-0.012
H5	1.294	-0.294	-0.878	-0.025

110-H3PO4

Atom	charge	net charge	diff charge	spin
P	1.353	3.647	4.224 (0.054)	0.002
O (PO)	7.421	-1.421	-0.253	0.014
O1 (OH)	7.400	-1.400	-0.232	0.000
H1 (OH)	0.384	0.616	0.032	0.000
O2 (OH)	7.400	-1.400	-0.232	0.000
H2 (OH)	0.377	0.623	0.039	0.000
O3 (OH)	7.354	-1.354	-0.186	0.000
H3 (OH)	0.354	0.646	0.062	0.000
H4	1.231	-0.231	-0.815	-0.018
H5	1.256	-0.256	-0.840	-0.013

O	7.283	-1.283	-0.115	0.060
O (OH)	7.312	-1.312	-0.144	-0.006
H (OH)	0.367	0.633	0.049	0.000
H1	1.323	-0.323	-0.907	-0.014
H2	1.343	-0.343	-0.927	-0.019
H3	1.324	-0.324	-0.908	-0.012

001-DEP2-POPOH

Atom	charge	net charge	diff charge	spin
P	4.488	0.512	1.083	-0.032
O	7.291	-1.291	-0.123	0.060
P (OH)	4.711	0.289	0.861	-0.057
O (OH)	7.304	-1.304	-0.136	-0.006
H (OH)	0.378	0.622	0.038	0.000
H1	1.328	-0.328	-0.912	-0.019
H2	1.320	-0.320	-0.904	-0.014
H3	1.314	-0.314	-0.898	-0.012

001-DEP3-POH

Atom	charge	net charge	diff charge	spin
P	4.688	0.312	0.883	-0.050
O1 (POH)	7.297	-1.297	-0.129	-0.011
H1 (POH)	0.403	0.597	0.013	0.005
O2 (OH)	7.226	-1.226	-0.058	0.077
H2 (OH)	0.366	0.634	0.050	0.005
O3 (OH)	7.221	-1.221	-0.053	0.082
H3 (OH)	0.379	0.621	0.037	0.007
H4	1.294	-0.294	-0.878	-0.015
H5	1.292	-0.292	-0.876	-0.016
H6	1.324	-0.324	-0.908	-0.018

H6	1.286	-0.286	-0.870	-0.017
H7	1.271	-0.271	-0.855	-0.028
H8	1.277	-0.277	-0.861	-0.012

001-DEP3-HPO3

Atom	charge	net charge	diff charge	spin
P	1.710	3.290	3.861 (-1.013)	0.010
H (PH)	1.396	-0.396	-0.068	0.000
O1	7.408	-1.408	-0.240	0.062
O2	7.384	-1.384	-0.216	0.050
O3	7.392	-1.392	-0.224	0.044
H1	1.280	-0.280	-0.864	-0.011
H2	1.315	-0.315	-0.899	-0.019
H3	1.281	-0.281	-0.865	-0.015
H4	1.323	-0.323	-0.907	-0.014
H5	1.289	-0.289	-0.873	-0.019

001-DEP3-HPO4

Atom	charge	net charge	diff charge	spin
P	1.363	3.637	4.209 (-0.956)	0.010
O (OH)	7.345	-1.345	-0.177	0.007
H (OH)	0.370	0.630	0.046	0.000
O1	7.400	-1.400	-0.232	0.077
O2	7.409	-1.409	-0.241	0.034
O3	7.380	-1.380	-0.212	0.068
H1	1.274	-0.274	-0.858	-0.010
H2	1.325	-0.325	-0.909	-0.020
H3	1.284	-0.284	-0.868	-0.015
H4	1.335	-0.335	-0.919	-0.013
H5	1.278	-0.278	-0.862	-0.021
H6	1.273	-0.273	-0.857	-0.012
H7	1.325	-0.325	-0.909	-0.019

001-DEP3-PO

Atom	charge	net charge	diff charge	spin
P	4.218	0.782	1.354	-0.050
O (PO)	7.272	-1.272	-0.104	-0.011
O1 (OH)	7.207	-1.207	-0.039	-0.057
H1 (OH)	0.371	0.629	0.045	0.005
O2 (OH)	7.203	-1.203	-0.035	0.082
H2 (OH)	0.378	0.622	0.038	0.007
H3	1.285	-0.285	-0.869	-0.015
H4	1.286	-0.286	-0.870	-0.016
H5	1.286	-0.286	-0.870	-0.018
H6	1.281	-0.281	-0.865	0.000

001-DEP4-2POH

Atom	charge	net charge	diff charge	spin
P1	4.654	0.346	0.917	-0.059
P2	4.660	0.340	0.911	-0.059
O1 (POH)	7.309	-1.309	-0.141	-0.013
H1 (POH)	0.399	0.601	0.017	0.005
O2 (POH)	7.284	-1.284	-0.116	-0.012
H2 (POH)	0.421	0.579	-0.005	0.005
O3 (OH)	7.205	-1.205	-0.037	0.083
H3 (OH)	0.390	0.610	0.026	0.005
O4 (OH)	7.240	-1.240	-0.072	0.082
H4 (OH)	0.356	0.644	0.060	0.005
H5	1.288	-0.288	-0.872	-0.016
H6	1.288	-0.288	-0.872	-0.016
H7	1.286	-0.286	-0.870	-0.016
H8	1.287	-0.287	-0.871	-0.016

001-DEP4-HPO2

001-DEP1

Atom	charge	net charge	diff charge	spin
O	7.228	-1.228	-0.060	0.072
H (OH)	0.381	0.619	0.035	0.006
H	1.346	-0.346	-0.930	-0.012

001-DEP2

Atom	charge	net charge	diff charge	spin
O	7.253	-1.253	-0.085	0.075
H (OH)	0.353	0.647	0.063	0.006
H	1.294	-0.294	-0.878	-0.015
O	7.218	-1.218	-0.050	0.075
H (OH)	0.389	0.611	0.027	0.006
H	1.294	-0.294	-0.878	-0.015

001-DEP3

Atom	charge	net charge	diff charge	spin
O	7.190	-1.190	-0.022	0.063
H (OH)	0.389	0.611	0.027	0.006
H	1.269	-0.269	-0.853	-0.013
O	7.211	-1.211	-0.044	0.068
H (OH)	0.385	0.615	0.031	0.004
H	1.282	-0.282	-0.866	-0.018
O	7.127	-1.127	0.041	0.083
H (OH)	0.429	0.571	-0.013	0.001
H	1.276	-0.276	-0.860	-0.026

Atom	charge	net charge	diff charge	spin
P	4.654	0.346	0.917	-0.028
O (PO)	7.284	-1.284	-0.116	0.054
O1 (POH)	7.309	-1.309	-0.141	-0.003
H1 (POH)	0.399	0.601	0.017	0.005
O2 (OH)	7.205	-1.205	-0.037	0.073
H2 (OH)	0.390	0.610	0.026	0.005
O3 (OH)	7.240	-1.240	-0.072	0.093
H3 (OH)	0.356	0.644	0.060	0.005
H4	1.288	-0.288	-0.872	-0.009
H5	1.288	-0.288	-0.872	-0.008
H6	1.286	-0.286	-0.870	-0.017
H7	1.287	-0.287	-0.871	-0.019
H8	0.421	0.579	-0.005	-0.009

001-DEP-TS

Atom	charge	net charge	diff charge	spin
O	7.091	-1.091	0.076	0.022
H (OH)	0.401	0.599	0.015	0.003
H	0.926	0.074	-0.510	-0.011

001-DEP-PROD

Atom	charge	net charge	diff charge	spin
O	7.136	-1.136	0.032	0.072
H (OH)	0.405	0.595	0.011	0.003
H	1.320	-0.320	-0.904	-0.016

Table S2: P—H stretching of H_3PO_3 and its deprotonated forms calculated at different levels of theory. Values are in cm^{-1}.

Species	H_3PO_3		$H_2PO_3^-$		HPO_3^{2-}	
Method	B2PLYP-D3BJ/ aug-cc-pvtz	PBE-D3BJ/ def2-QZVPP	B2PLYP-D3BJ/ aug-cc-pvtz	PBE-D3BJ/ def2-QZVPP	B2PLYP-D3BJ/ aug-cc-pvtz	PBE-D3BJ/ def2-QZVPP
Harmonic	2560	2437	2332	2196	1962	1781
Anharmomic	2463	2339	2221	2074	1747	1608
Diff (B2PLYP/Anharm - PBE/Harm)	27		25		-34	

<table>
<tr>
<td>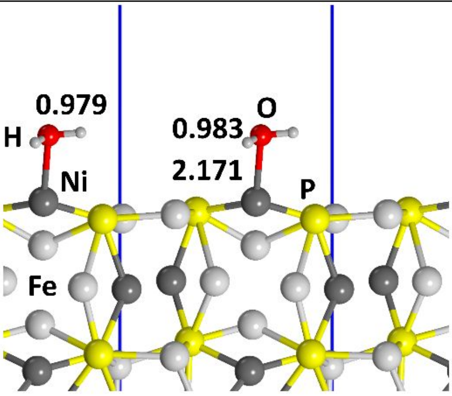
</td>
<td>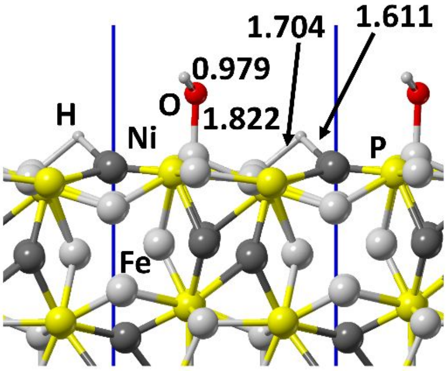
</td>
</tr>
<tr>
<td>110-MOL1 (0.0) [-37.6]</td>
<td>110-DEP1 (29.1) [-12.5]</td>
</tr>
</table>

Figure S1: PBE-D*0 optimized structures of water adsorbed on the (110) Fe_2NiP surface. Energy values in kJ/mol. In round parenthesis the 110-MOL1 is the reference, in square parenthesis the adsorption energy with respect the free reactants. Atom color legend: H in white, O in red, P in yellow, Fe in light grey, Ni in dark grey.

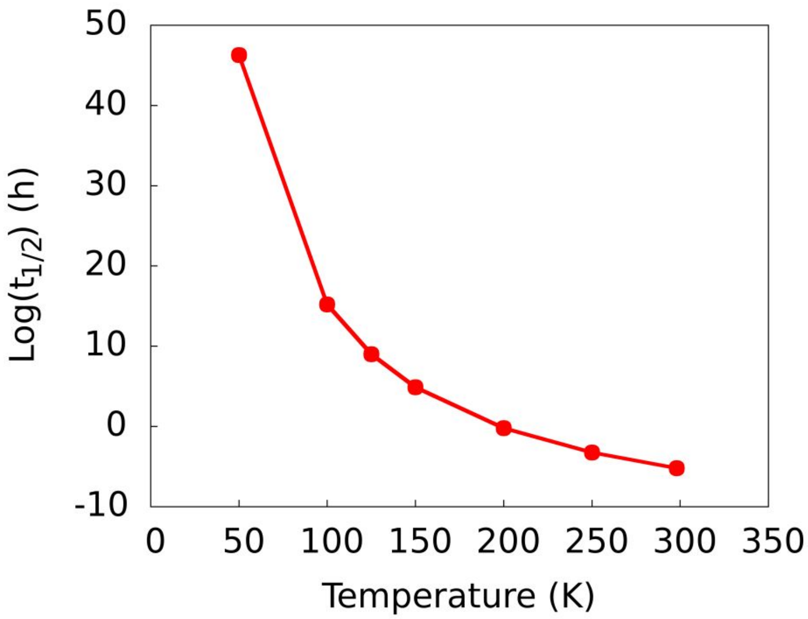

Figure S2: Logarithm of the half-life time ($Log(t_{1/2})$, in hours) vs Temperature (in K).

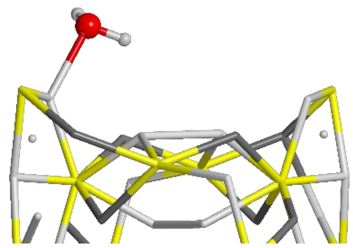	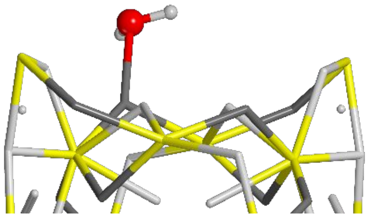
a) 001-MOL-R (-63.7)	b) 001-MOL-Ni1 (-38.9)
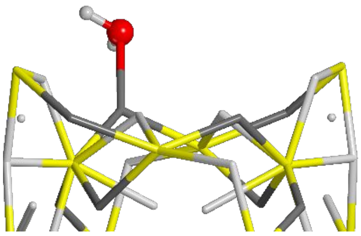	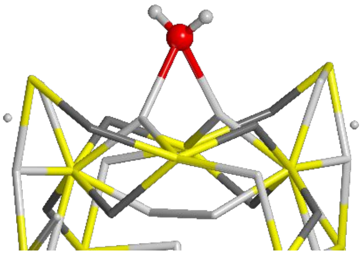
c) 001-MOL-Ni2 (-38.2)	d) 001-MOL-FeFe (-33.0)

Figure S3: PBE-D*0 optimized structures of molecular water adsorbed on the (001) Fe_2NiP surface. Adsorption energy values in kJ/mol. Atom color legend: H in white, O in red, P in yellow, Fe in light grey, Ni in dark grey.

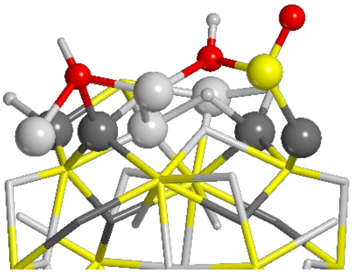	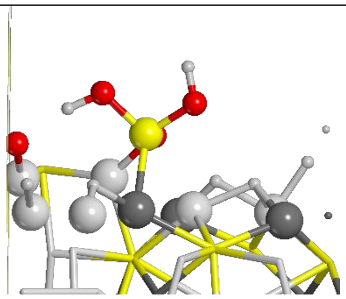
001-DEP3-POPOH **(-61.4)**	001-DEP4-H2PO3 **(-24.1)**
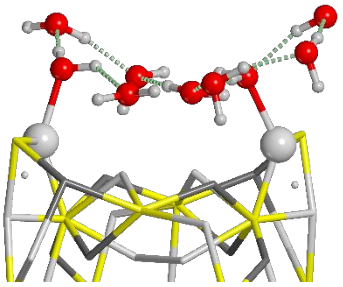	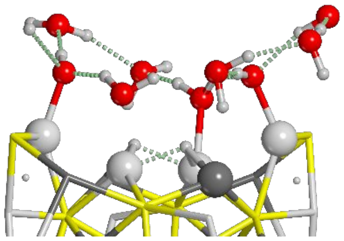
001-MONO **(62.3)**	001-MONO-DEP **(59.6)**

Figure S4: PBE-D*0 optimized structures of deprotonated water adsorbed on the (001) Fe_2NiP surface (top) and molecular and deprotonated water monolayer (bottom). Adsorption energy values in kJ/mol, for the monolayer the adsorption energies are normalized per single water molecule. Atom color legend: H in white, O in red, P in yellow, Fe in light grey, Ni in dark grey.

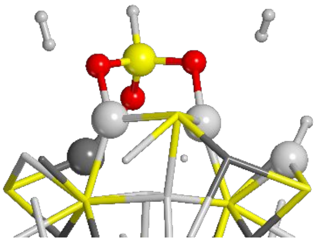	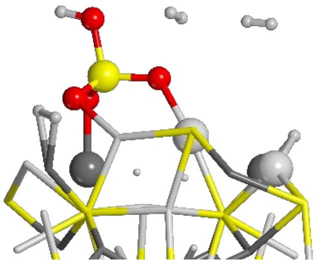
a) 001-HPO3-H2 (57.9)	b) 001-HPO4-H2 (56.4)

Figure S5: PBE-D*0 optimized structures of HPO_3^{2-} and HPO_4^{2-} with physisorbed H_2 molecules on the (001) Fe_2NiP surface. Reaction energy values in kJ/mol, with respect to the reactants (001-HPO3-R and 001-HPO4-R). Atom color legend: H in white, O in red, P in yellow, Fe in light grey, Ni in dark grey.

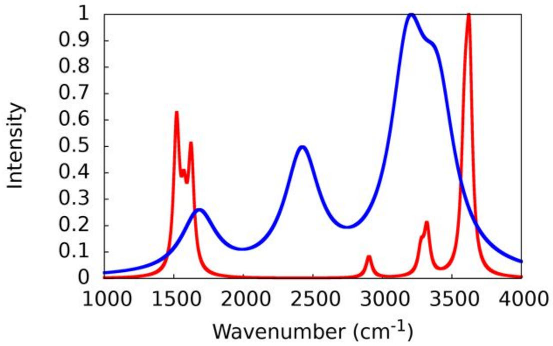

a) 001-HPO3 reactant, gas phase

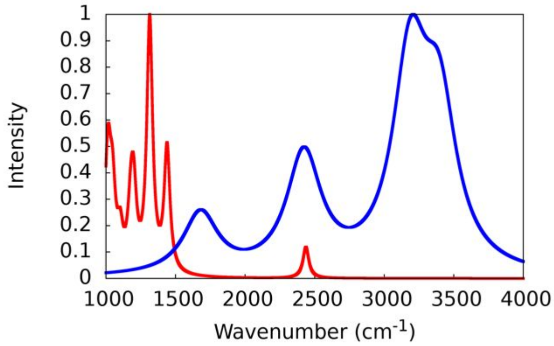

b) 001-HPO3 product, gas phase

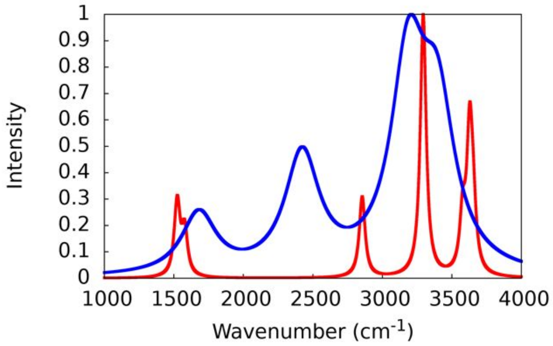

c) 001-HPO3 reactant, PCM

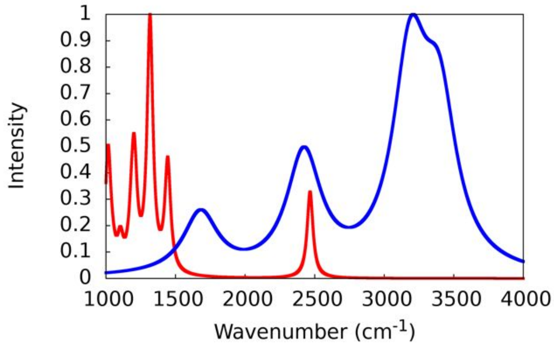

d) 001-HPO3 product, PCM

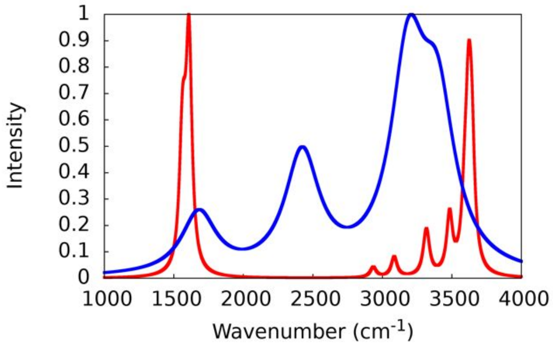

e) 001-HPO4 reactant, gas phase

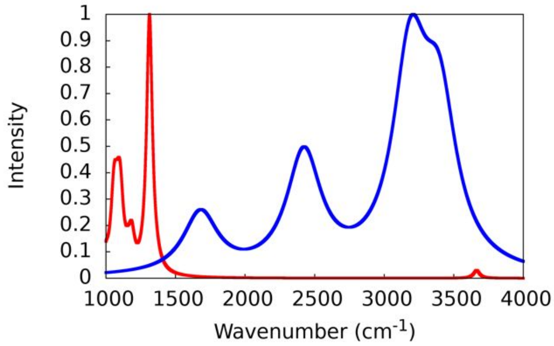

f) 001-HPO4 product, gas phase

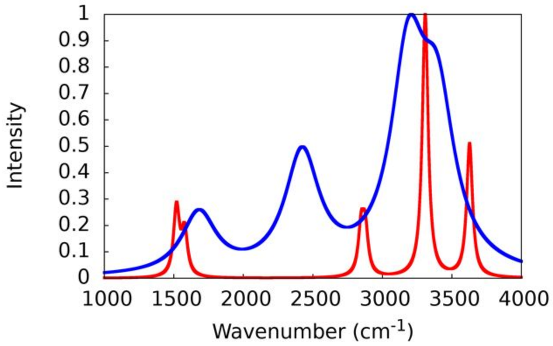

g) 001-HPO4 reactant, PCM

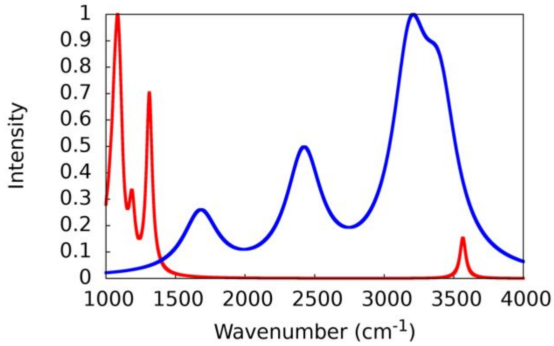

h) 001-HPO4 product, PCM

Figure S6: PBE-D*0 simulated spectra of 001-HPO3 and 001-HPO4 reactants and products on the (001) Fe_2NiP surface.

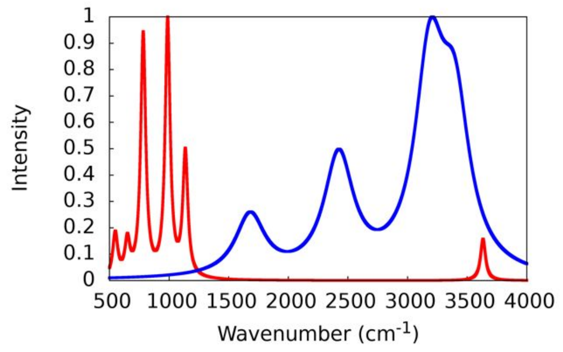	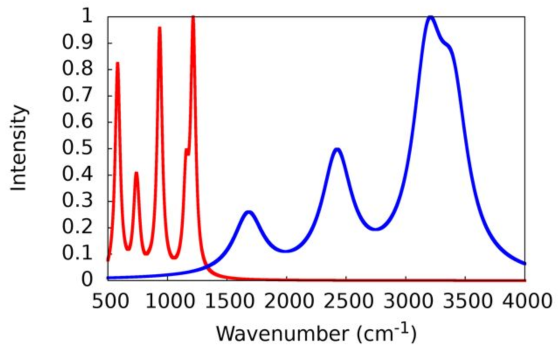
a) 001-DEP1	b) 001-DEP1-POFe
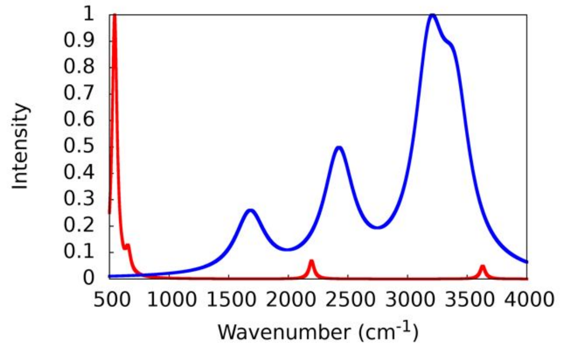	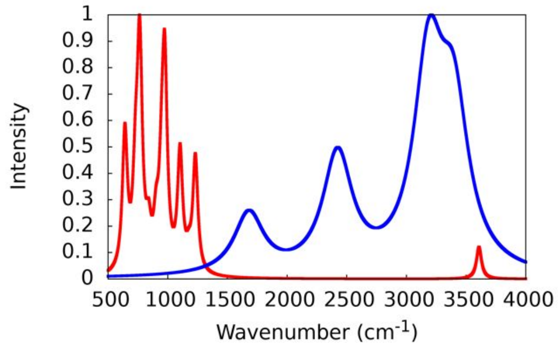
c) 001-DEP1-PH	d) 001-DEP2-HPO2

Figure S7: PBE-D*0 simulated spectra of water deprotonated structures on the (001) Fe_2NiP surface.

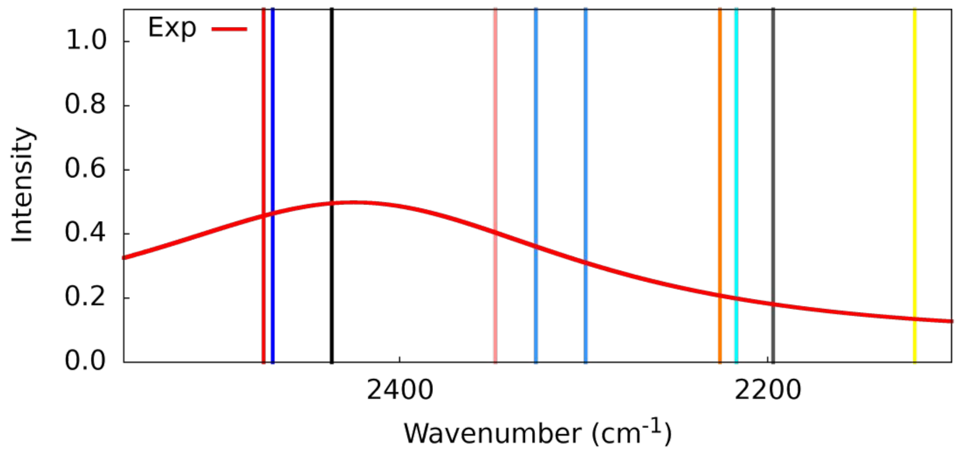		
a) IR spectrum		
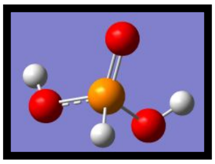	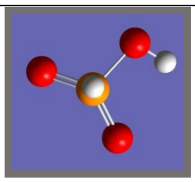	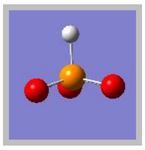
b) H_3PO_3 (2437)	c) $H_2PO_3^-$ (2197)	d) HPO_3^{2-} (1781)
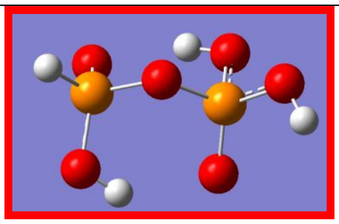	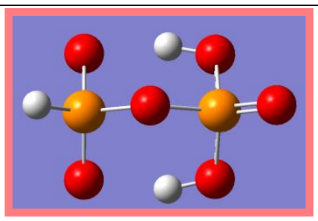	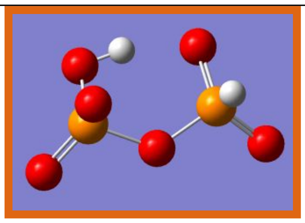
e) $H_4P_2O_6$ (2474)	f) $H_3P_2O_6^-$ (2348)	g) $H_2P_2O_6^{2-}$ (2226)
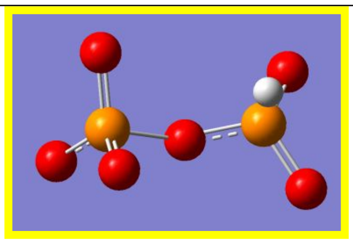	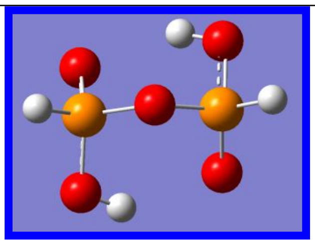	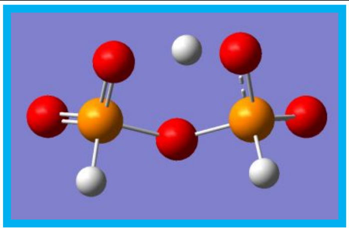
h) $HP_2O_6^{3-}$ (2120)	i) $H_4P_2O_5$ (2469)	j) $H_3P_2O_5^-$ (2299-2326)
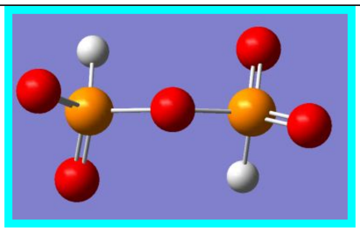		
k) $H_2P_2O_5^{2-}$ (2217)		

Figure S8: a) PBE-D2/def2-QZVPP P—H stretching (colored bars) and from b) to k) optimized structures of H_3PO_3, $H_4P_2O_6$, $H_4P_2O_5$, and their corresponding deprotonated forms. In parenthesis the P—H stretching in cm^{-1}.

<table>
<tr>
<td>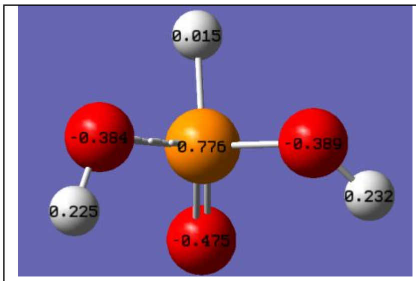
</td>
<td>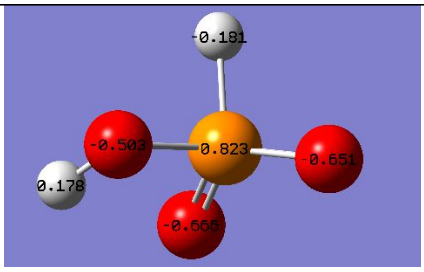
</td>
<td>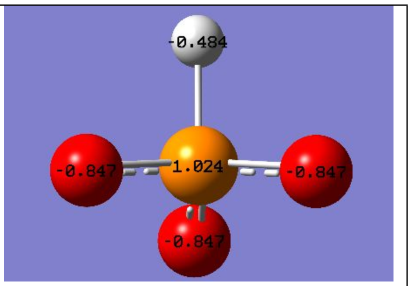
</td>
</tr>
<tr>
<td>a) H_3PO_3</td>
<td>b) $H_2PO_3^-$</td>
<td>c) HPO_3^{2-}</td>
</tr>
</table>

Figure S9: PBE-D2/def2-QZVPP optimized structures of H_3PO_3 and its deprotonated derivatives. The numbers on the atoms correspond to the Mulliken charges.

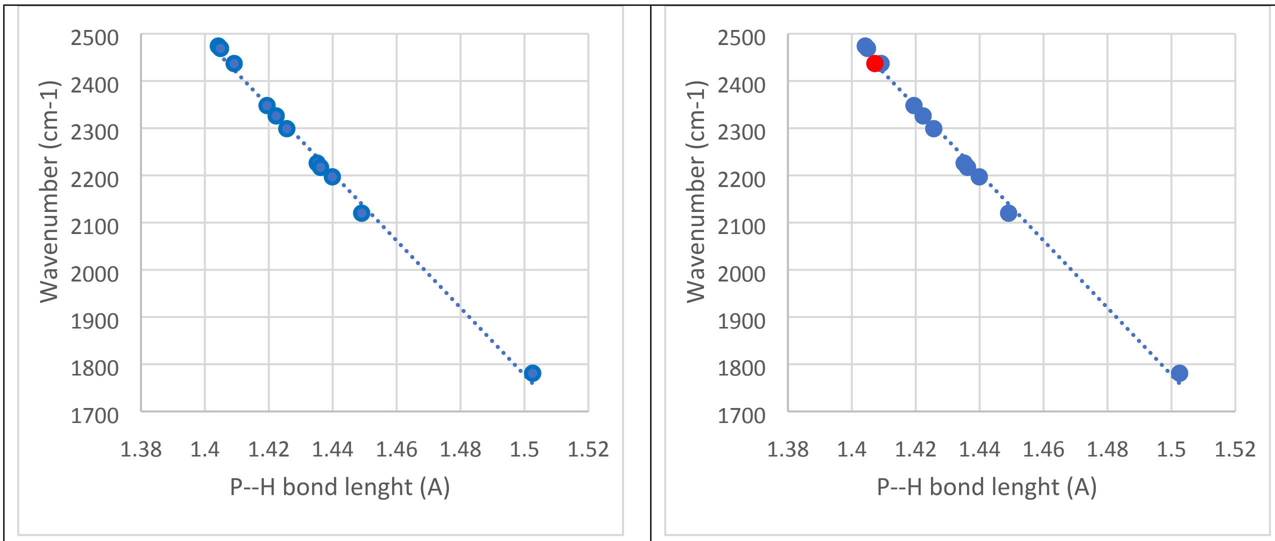

a) Gaussian	b) Gaussian + VASP

Figure S10: Correlation between stretching frequency and P—H bond length at PBE level. a) Gaussian results only, and b) Gaussian + VASP (red dot) results.

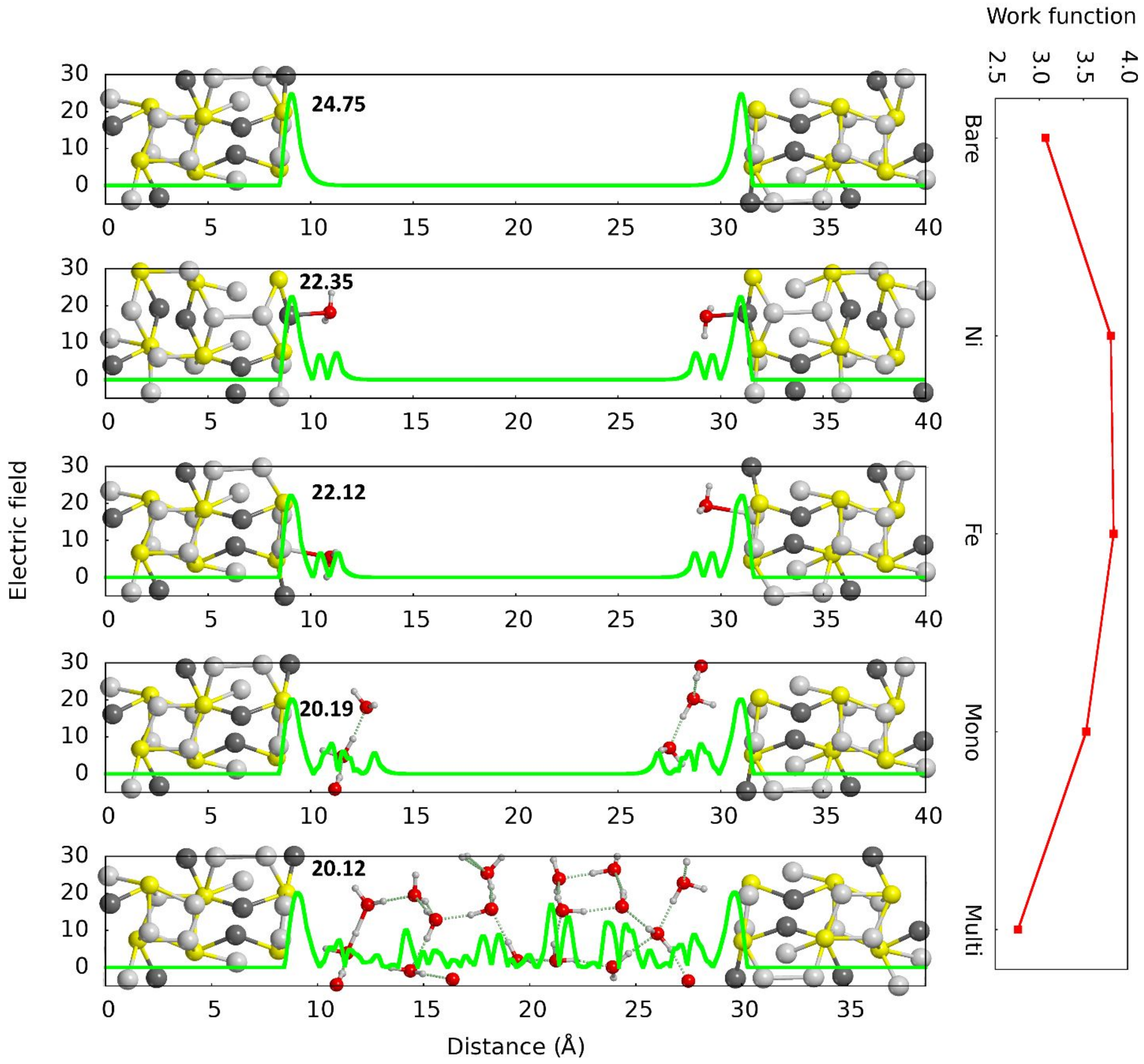

Figure S11: Electric field (in V/Å) and work function (in eV) of the different molecular and reactive water adsorption models on the (110) Fe_2NiP. Atom color legend: H in white, O in red, P in yellow, Fe in light grey, Ni in dark grey.

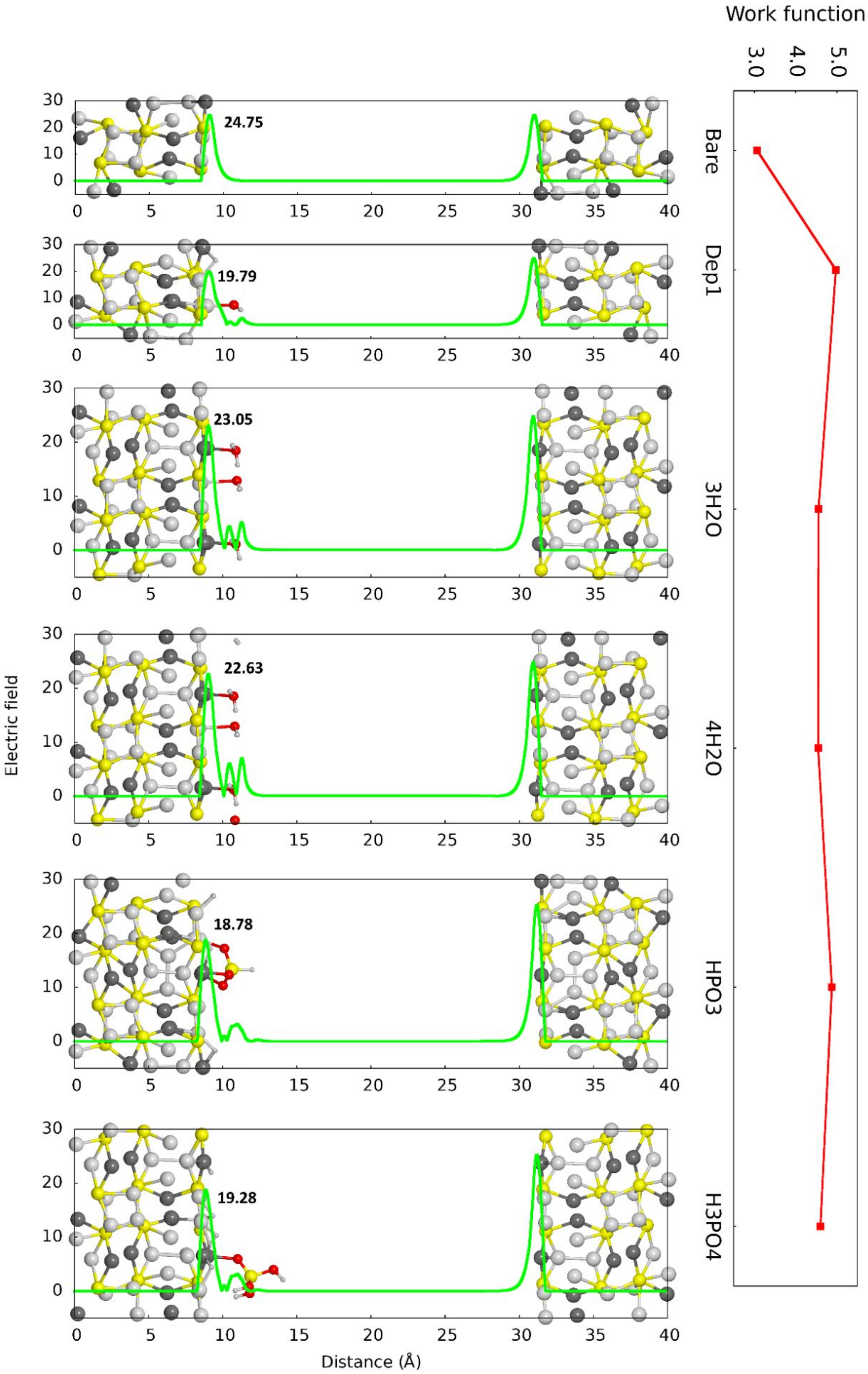

Figure S12: Electric field (in V/Å) and work function (in eV) of the different molecular and reactive water adsorption models on the (110) Fe_2NiP. Atom color legend: H in white, O in red, P in yellow, Fe in light grey, Ni in dark grey.

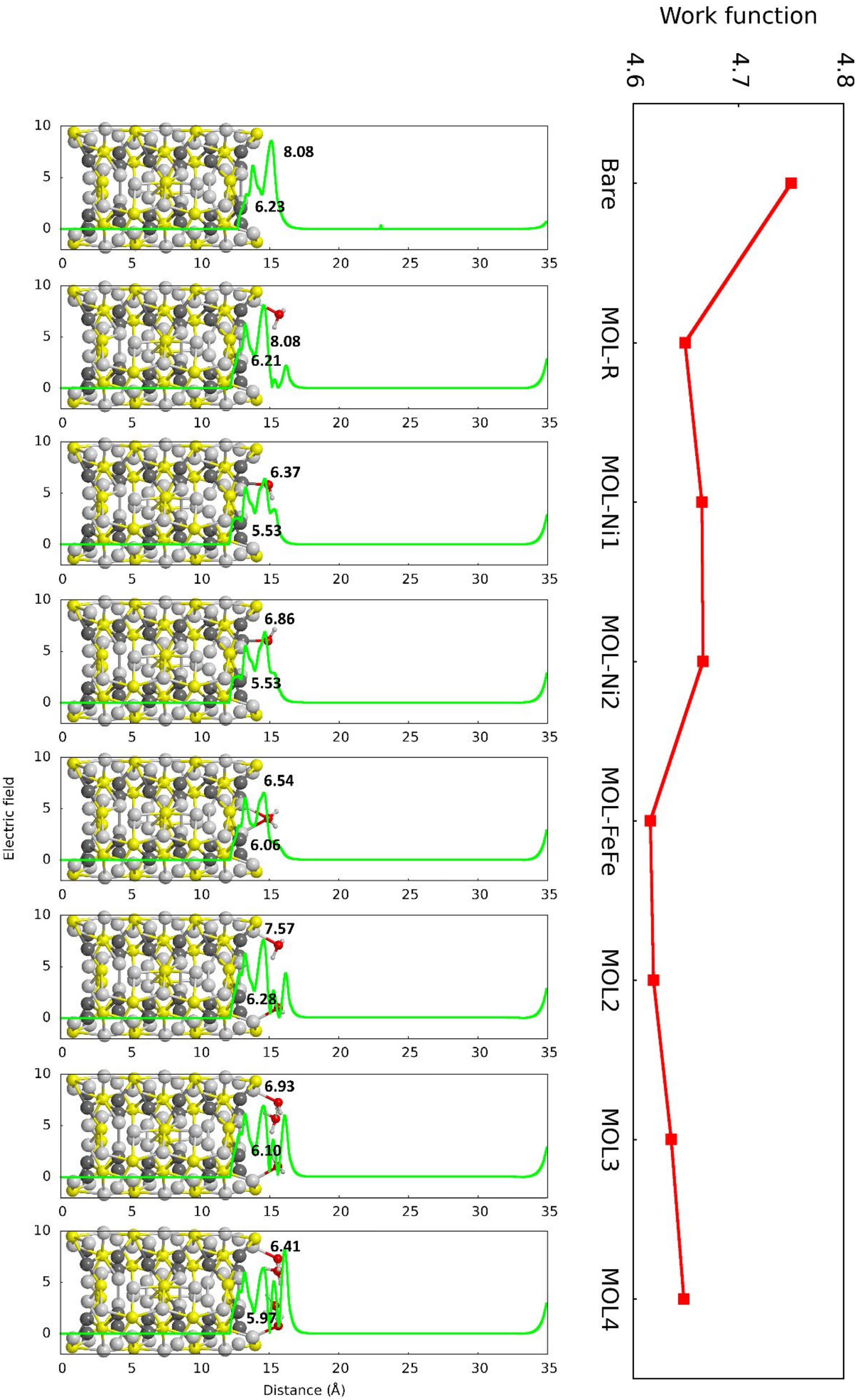

Work function
4.6
4.7
4.8
Bare
MOL-R
MOL-Ni1
MOL-Ni2
MOL-FeFe
MOL2
MOL3
MOL4
Electric field
Distance (Å)
8.08
6.23
8.08
6.21
6.37
5.53
6.86
5.53
6.54
6.06
7.57
6.28
6.93
6.10
6.41
5.97

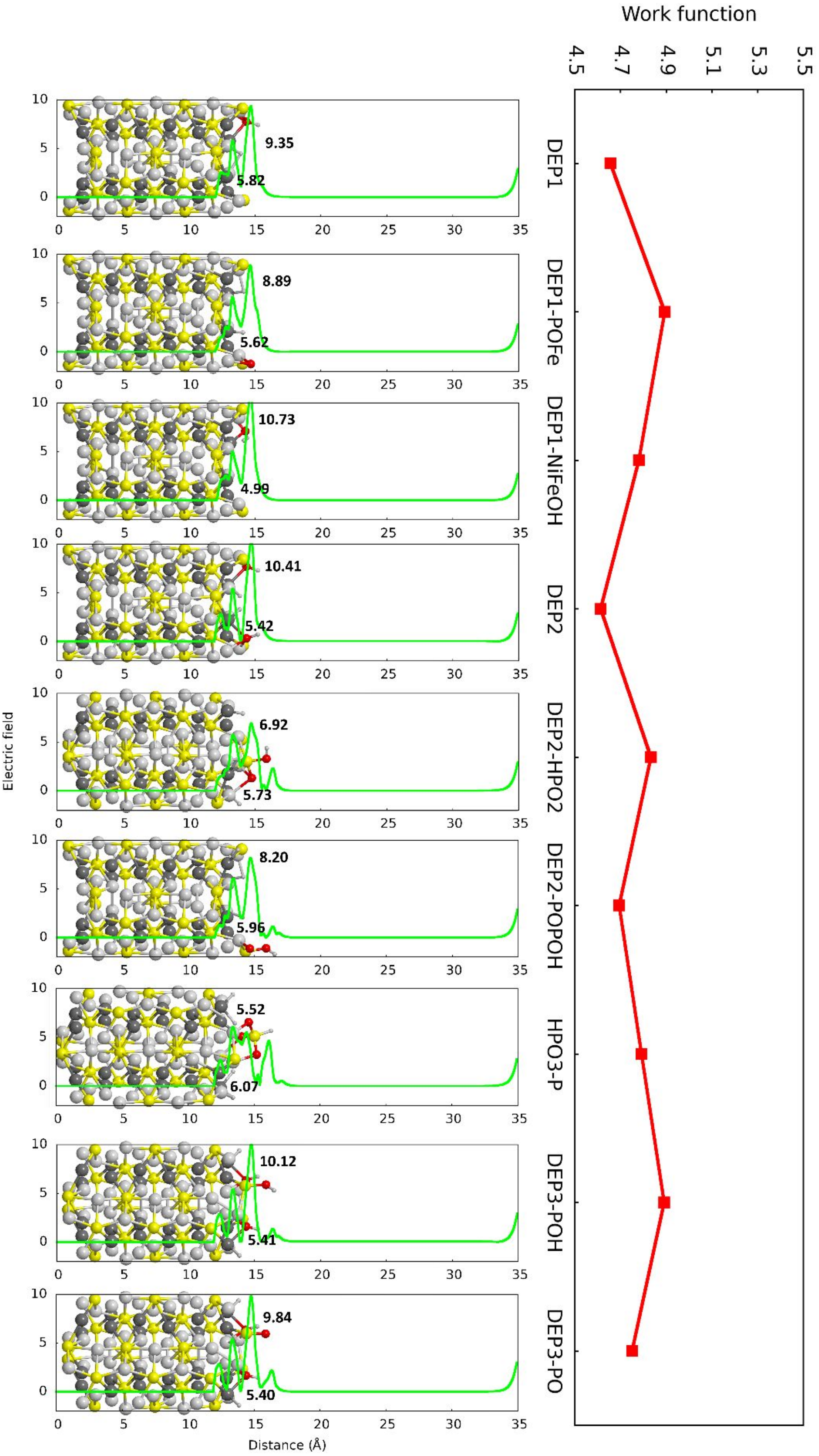

Work function
4.5
4.7
4.9
5.1
5.3
5.5
DEP1
DEP1-POFe
DEP1-NiFeOH
DEP2
DEP2-HPO2
DEP2-POPOH
HPO3-P
DEP3-POH
DEP3-PO
9.35
5.82
8.89
5.62
10.73
4.99
10.41
5.42
6.92
5.73
8.20
5.96
5.52
6.07
10.12
5.41
9.84
5.40
Electric field
Distance (Å)

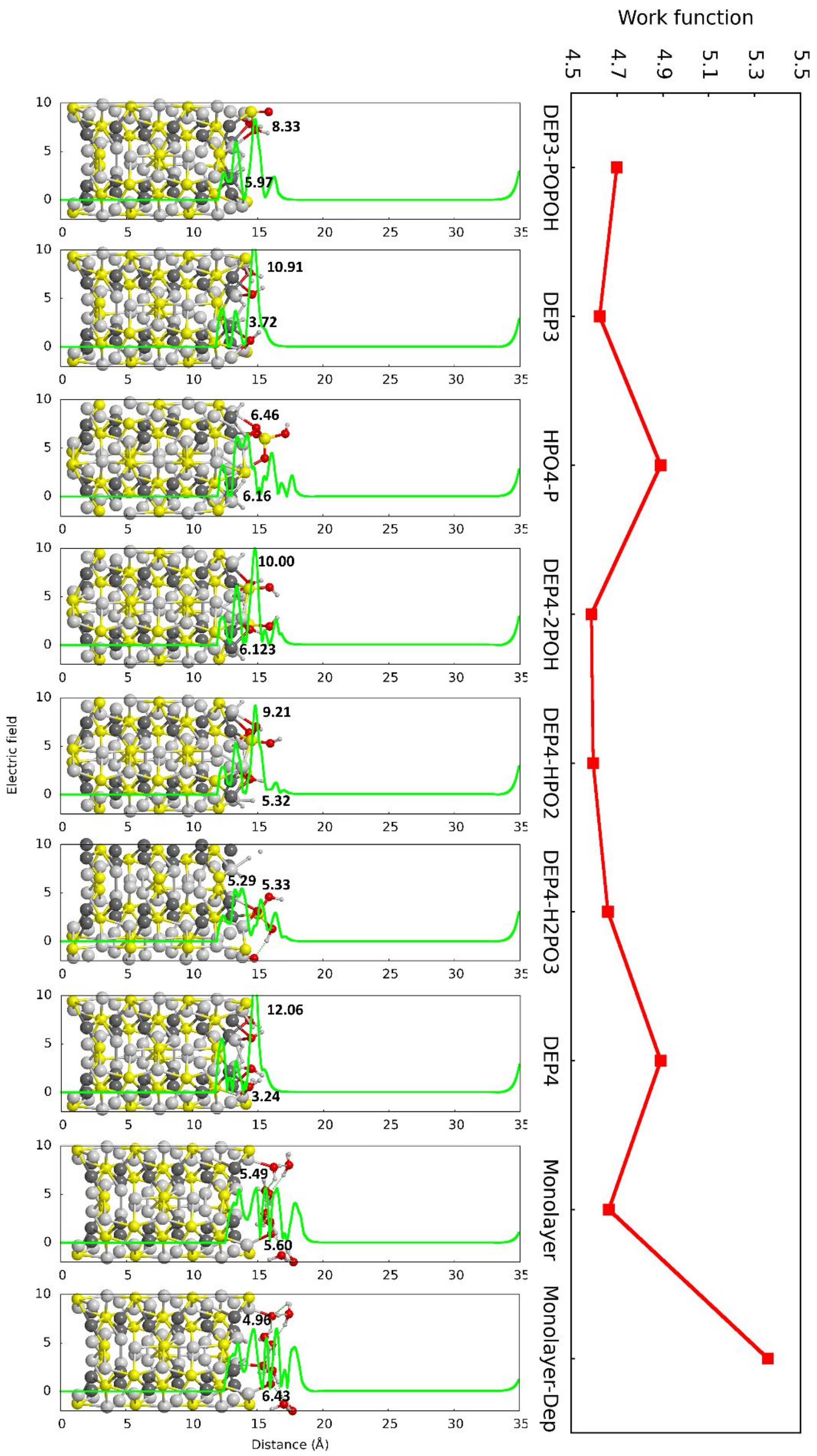

Figure S13: Electric field (in V/Å) and work function (in eV) of molecular and deprotonated water adsorption models on the (001) Fe_2NiP. Atom color legend: H in white, O in red, P in yellow, Fe in light grey, Ni in dark grey.

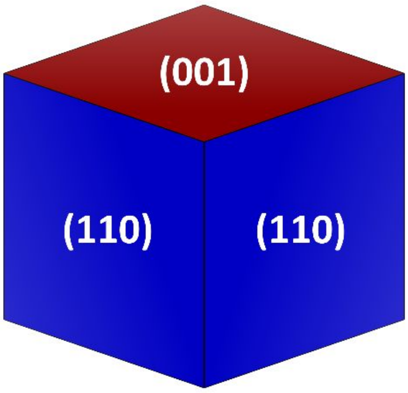

a) 0 K: 64.9 % for (110), 35.1 % for (001)

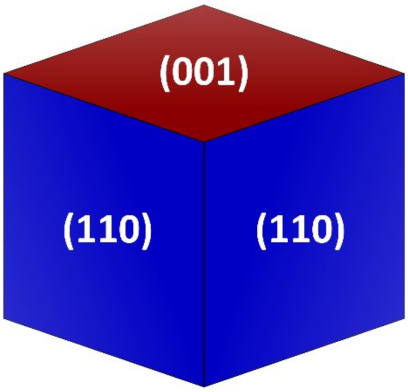

b) 125 K: 64.7 % for (110), 35.3 % for (001)

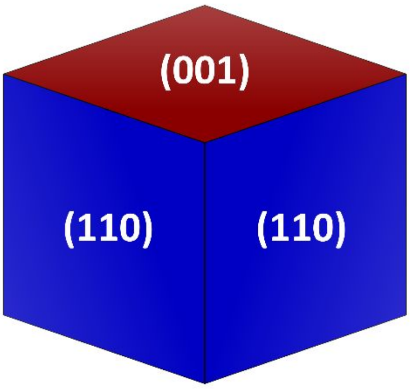

c) 298 K: 64.3 % for (110), 35.7 % for (001)

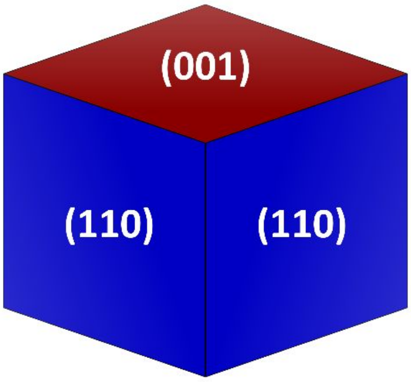

d) 600 K: 63.5 % for (110), 36.5 % for (001)

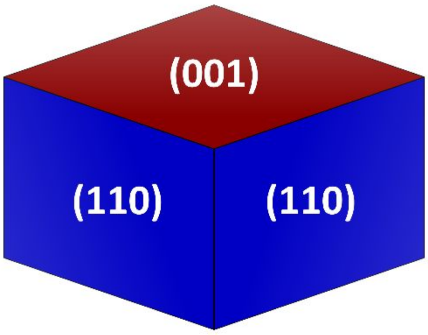

e) 3000 K: 56.4 % for (110), 43.6 % for (001)

Figure S14: Wulff polyhedron, calculated at PBE-D*0/500 eV level of theory as a function of the temperature. (110) surface in blue, (001) in red.

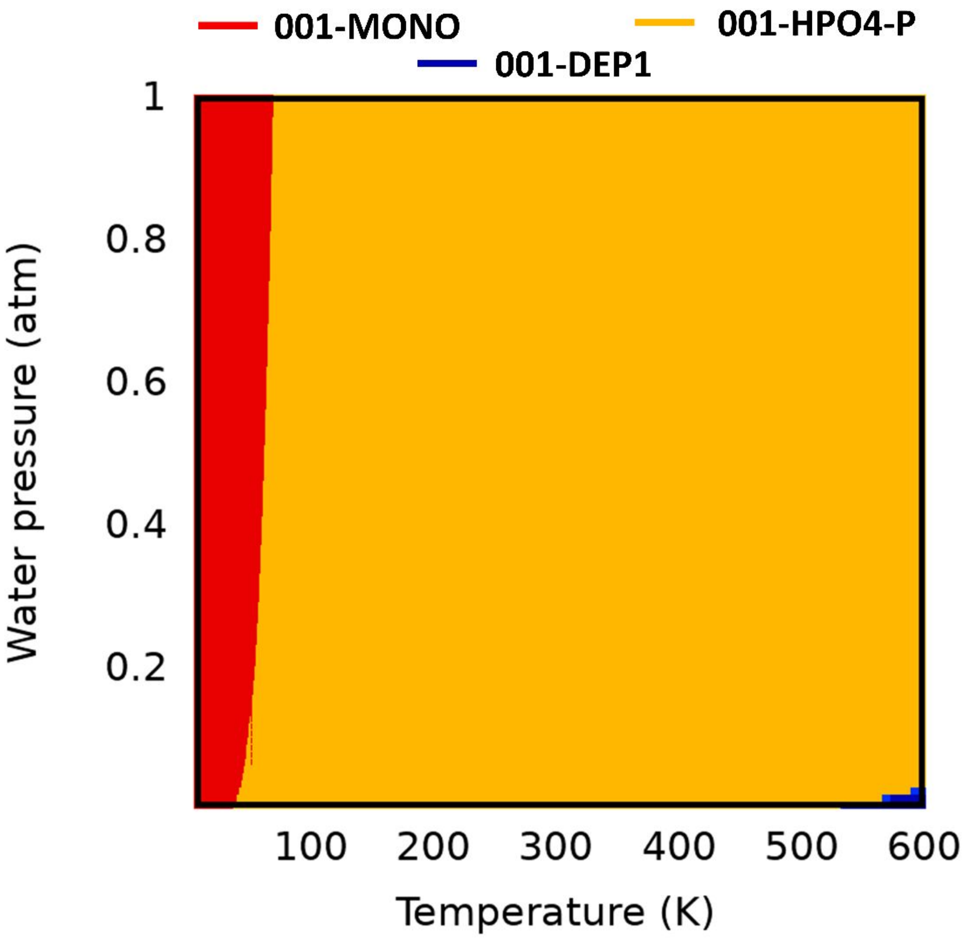

Figure S15: Phase diagram of all water adsorptions (molecular and deprotonated) on the (001) Fe_2NiP surface as a function of temperature and water pressure.

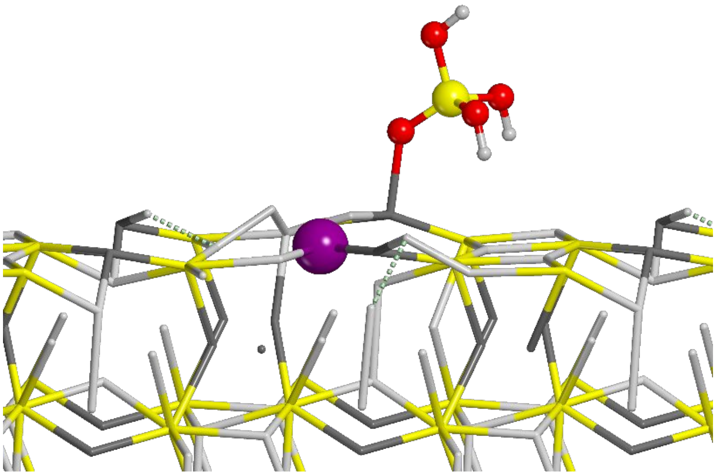

Figure S16: PBE-D*0 optimized structures of 110-H3PO4. The purple sphere corresponds to the previous P atom position, when it was part of the surface. Atom color legend: H in white, O in red, P in yellow, Fe in light grey, Ni in dark grey.
